# Impaired lysosomal activity mediated autophagic flux disruption by graphite carbon nanofibers induce apoptosis in human lung epithelial cells through oxidative stress and energetic impairment

**DOI:** 10.1186/s12989-017-0194-4

**Published:** 2017-04-28

**Authors:** Sandeep Mittal, Pradeep Kumar Sharma, Ratnakar Tiwari, Raja Gopal Rayavarapu, Jai Shankar, Lalit Kumar Singh Chauhan, Alok Kumar Pandey

**Affiliations:** 1 Academy of Scientific and Innovative Research (AcSIR), CSIR-IITR Campus, Lucknow, India; 2 Nanomaterials Toxicology Laboratory, Nanotherapeutics and Nanomaterial Toxicology Group, CSIR - Indian Institute of Toxicology Research (CSIR - IITR), Vishvigyan Bhawan, 31, Mahatma Gandhi Marg, Lucknow, 226001 Uttar Pradesh India; 3 Environmental Carcinogenesis Laboratory, Food, Drug and Chemical Toxicology Group, CSIR - Indian Institute of Toxicology Research (CSIR - IITR), Vishvigyan Bhawan, 31, Mahatma Gandhi Marg, Lucknow, 226001 Uttar Pradesh India; 4 Developmental Toxicology Laboratory, System Toxicology and Health Risk Assessment Group, CSIR - Indian Institute of Toxicology Research (CSIR - IITR), Vishvigyan Bhawan, 31, Mahatma Gandhi Marg, Lucknow, 226001 Uttar Pradesh India; 5 Electron Microscopy Laboratory, CSIR - Indian Institute of Toxicology Research (CSIR - IITR), Vishvigyan Bhawan 31, Mahatma Gandhi Marg, Lucknow, 226001 Uttar Pradesh India

**Keywords:** Graphite carbon nanofibers (GCNF), Autophagy, Apoptosis, Mitochondrial damage, Destabilization of lysosomes, ATP loss, Reactive oxygen species (ROS), Genotoxicity

## Abstract

**Background:**

Graphite carbon nanofibers (GCNF) have emerged as a potential alternative of carbon nanotubes (CNT) for various biomedical applications due to their superior physico-chemical properties. Therefore in-depth understanding of the GCNF induced toxic effects and underlying mechanisms in biological systems is of great interest. Currently, autophagy activation by nanomaterials is recognized as an emerging toxicity mechanism. However, the association of GCNF induced toxicity with this form of cell death is largely unknown. In this study, we have assessed the possible mechanism; especially the role of autophagy, underlying the GCNF induced toxicity.

**Methods:**

Human lung adenocarcinoma (A549) cells were exposed to a range of GCNF concentrations and various cellular parameters were analyzed (up to 48 h). Transmission electron microscopy, immunofluorescent staining, western blot and quantitative real time PCR were performed to detect apoptosis, autophagy induction, lysosomal destabilization and cytoskeleton disruption in GCNF exposed cells. DCFDA assay was used to evaluate the reactive oxygen species (ROS) production. Experiments with N-acetyl-L-cysteine (NAC), 3-methyladenine (3-MA) and LC3 siRNA was carried out to confirm the involvement of oxidative stress and autophagy in GCNF induced cell death. Comet assay and micronucleus (MN) assay was performed to assess the genotoxicity potential.

**Results:**

In the present study, GCNF was found to induce nanotoxicity in human lung cells through autophagosomes accumulation followed by apoptosis via intracellular ROS generation. Mechanistically, impaired lysosomal function and cytoskeleton disruption mediated autophagic flux blockade was found to be the major cause of accumulation rather than autophagy induction which further activates apoptosis. The whole process was in line with the increased ROS level and their pharmacological inhibition leads to mitigation of GCNF induced cell death. Moreover the inhibition of autophagy attenuates apoptosis indicating the role of autophagy as cell death process. GCNF was also found to induce genomic instability.

**Conclusion:**

Our present study demonstrates that GCNF perturbs various interrelated signaling pathway and unveils the potential nanotoxicity mechanism of GCNF through targeting ROS-autophagy-apoptosis axis. The current study is significant to evaluate the safety and risk assessment of fibrous carbon nanomaterials prior to their potential use and suggests caution on their utilization for biomedical research.

**Electronic supplementary material:**

The online version of this article (doi:10.1186/s12989-017-0194-4) contains supplementary material, which is available to authorized users.

## Background

Carbon, being the most abundant material in nature has varied forms of allotropes that are structurally/chemically different from one another leading to different functional materials at nanoscale [1]. Nanotechnology has evolved to generate various unique carbon based nanomaterials (CBNM) such as fullerenes, graphene, carbon nanotubes (CNT) and graphite carbon nanofibers (GCNF), that have garnered increasing interest in both technological advances and manufacturing materials for end-consumer needs [1]. Critical factors influencing the potential of these materials are their surface properties and the structural orientations. The unique nature of tubular structure in fibrous carbon nanomaterials (CNT and GCNF) make them potential candidate for various applications.

Among existing CBNM, GCNF have emerged as attractive nanomaterial (NM) due to their unique absorption and storage capacity, flexibility, mechanical strength, ease of functionalization and low cost of production compared to CNT [2]. Despite GCNF and CNT belonging to the same carbon family and having resemblance in structure, their physico-chemical properties (shape, size, dispersion, durability) are different leading to their unique end-applications. GCNF have unique morphology that are made up of stacked graphene nano-cones with exposed edge planes on the interior and exterior surfaces of the fiber [3] which enables them to enhance their physico-chemical properties. These materials due to the orientation of functional molecules on their surfaces become ideal candidates for biomedical applications and to an extent biocompatible to health-care and biosensing applications [4, 5]. Further GCNF mimics to the physiological molecules (protein and DNA) and has the ability to be modified with different bioactive molecules which can be harnessed for tissue regeneration and for treating several pathologies [6]. Other striking feature is the aspect ratio of these materials that helps them to act as potential clinical scaffolds for neural tissue engineering [7, 8], bone regeneration [9] and regenerative medicine [10]. In addition to these biomedical applications, GCNF are widely employing as electrodes [11], catalyst support [12], absorbents for organic molecules [13] and for storage of hydrogen gases [14].

Despite the widespread use, there is dearth about GCNF interaction with biological systems compared to CNT. Notably, in spite of being a member of same family, GCNF has received partial attention of the scientific community in terms of their toxicity evaluation. In occupational settings, aerosol formation of fibrous carbon nanomaterials (CNT and GCNF) is quite evident in workplace rendering worker to get exposed to them [15]. In fact, due to very small in size, these biopersistant NM retain in the air for considerable amount of time and comes under the respirable limit (>15nm) of nanofibers [16]. Lung alveolar region is the prime site of deposition for inhaled fibrous nanomaterials. Upon inhalation, fibrous carbon nanomaterials can be deposited in respiratory tract for considerable duration that could result in altered lung physiology. Including this, the dimensions of fibrous carbon nanomaterials resemble to naturally occurring asbestos fiber, a potential carcinogen [17] which further warrants their safety evaluation. Previously, upon interaction CNT has been shown to be deposited in respiratory tract for considerable duration which results in alteration of lung functions and systemic toxicity [18–22]. However, less information is available regarding GCNF responses [23, 24] and mechanistic role towards biological cells is still unknown. Therefore, the present study has been conceived to understand the GCNF interaction with human lung cell to simulate conditions related to occupational exposure owing to the fact of their resemblance to CNT and asbestos. An evaluation of such interactions of GCNF becomes even more pertinent due to their increased potential nanomedicine applications.

Recent reports have suggested the activation of autophagy as well as apoptotic cell death including autophagy together with apoptosis after NM exposure [25, 26]. Autophagy is a highly regulated catabolic process which sequestered damaged organelles or unnecessary proteins, via their degradation through lysosomes and known to be involved in development, tissue remodeling and cytoprotective mechanism under adverse conditions [27]. Contrary to this, autophagic cell death has also been published upon exposure of NM including CNT [28–31]. On the other side, apoptosis is an energy dependent type I programmed cell death which induced either by pathological or physiological stimuli resulted into a cell death [32]. Although these two modes of cell death are distinct form but there boundaries are not properly demarcated. To regulate the cell survival and death both autophagy and apoptosis works in a co-ordinate manner [28, 33] and there is significant cross-talk exists [34]. Till now the interaction of GCNF with these forms of cell death is unknown with underlying molecular mechanism. Thus, a thorough understanding of GCNF interaction with these forms of cell death can help to formulate the safe and consumer friendly nano-products along with better strategies for various human disorders.

In the current study, we have investigated the interaction of GCNF and demonstrate the underlying molecular mechanism in in vitro human lung adenocarcinoma (A549) cells, using as a pulmonary like cell system. Here, the involvement of AMPK/mTOR mediated autophagic flux disruption and mitochondrial damage mediated apoptosis pathway via decline in ATP level was found following GCNF exposure. In addition, the autophagy flux was found to be halted through the decreased activity of lysosomes and cytoskeleton disruption which turning on the apoptotic cell death pathway. Interestingly, inhibition of GCNF induced autophagy results into the decreased level of apoptosis confirming its role as cell death pathway. The complete process was governed by the elevated level of reactive oxygen species (ROS) which was further confirmed by the decreased autophagy and apoptosis after inhibition of ROS. Our results strongly suggest that GCNF possess capacity to affect lung physiology and induce autophagosomes accumulation mediated apoptosis via the induction of oxidative stress and destabilization of lysosomes. Also, our data may shed light for developing the GCNF based biomedical applications to treat the diseases related to disturbance in the autophagic process.

## Methods

### Chemicals and reagents

Graphite carbon nanofibers (GCNF) powder (purity 95%, O.D. x I.D. x L – 200–500 x 1–10 nm x 10 – 40 μm), 3-[4,5-dimethylthiazol-2-yl]-2,5-diphenyltetrazolium bromide (MTT), Propidium Iodide (PI), 5,5’, 6,6’-Tetrachloro-1,1’,3,3’-tetraethyl- benzimidazolecarbocyanine iodide (JC-1) dye, paraformaldehyde (PFA), Dimethyl sulfoxide (DMSO), 2′,7′-Dichlorofluorescein diacetate (DCFDA), chloroquine, Monodansyl cadaverine (MDC), wortmanin, chlorpromazine, amiloride and anti - SQSTM1 primary antibody were procured from Sigma-Aldrich (St. Louis, Missouri, USA). Phosphate buffered saline (Ca^+2^, Mg^+2^ free; PBS), Dulbecco’s modified eagle medium : nutrient mixture F-12 (Ham) (1 : 1) powder (DMEM F-12), trypsin - EDTA, foetal bovine serum (FBS), antibiotic and antimycotic solution (10,000 U/ml penicillin, 10 mg/ml streptomycin, 25 μg/ml amphotericin-B), secondary antibodies conjugated to HRP/Alexa 488/Alexa 568, lysotracker red, Oregon green 488 phalloidin, pHrodo Green Dextran were purchased from Life Technologies Pvt. Ltd (Invitrogen, Carlsbad, CA, USA). Primary antibodies such as anti-β-actin, anti-beclin-1, anti-mTOR, and anti-p-mTOR were obtained from Cell Signaling Technology Inc. (Danvers, MA, USA). Luminescent ATP detection assay kit, anti-caspase-3, anti-PARP-1, anti-cyt C, anti-GAPDH were procured from Abcam (Cambridge, UK). Anti-LC3 antibody was procured from Novus Biologicals (Littleton, CO, USA). GFP - LC3 Plasmid and Mito - DsRed plasmid were kind gift from Dr. Soumya Sinha Roy, CSIR - IGIB, India. LC3 siRNA was purchased from Santa Cruz Biotechnology Inc. (Dallas, Texas, USA). Antifade mounting media Vectashield was purchased from Vector Laboratories Inc (Burlingame, CA, USA). Real time primers for various genes were designed and purchased from Integrated DNA technologies (IDT), (Coralville, Iowa, USA). Formamidopyrimidine DNA glycosylase (Fpg) Enzyme was obtained from Trevigen, Inc., USA.

## Characterization of GCNF

### Preparation of GCNF dispersion

For the characterization purpose, stock suspension of GCNF (150 μg/ml) was prepared by re-suspending them in Milli-Q water as well as in DMEM F-12 culture medium supplemented with 10% FBS. Then to reduce the agglomerates, suspension was subjected to probe sonication (Sonics Vibra cell, Sonics & Material Inc., New Town, CT, USA) at 30 W for total 10 min (2.5 min pulse on and 1 min pulse off for 4 times) and allow to cool. Further different concentrations ranging from 1 to 100 μg/ml was prepared using stock solution and DMEM F-12 culture medium supplemented with 10% FBS.

### Dynamic light scattering (DLS)

The average hydrodynamic size, size distribution and zeta potential of GCNF were determined in culture medium supplemented with 10% FBS by employing dynamic light scattering (DLS) and phase analysis light scattering respectively using a ZetasizerNano-ZS equipped with 4.0 mW, 633 nm laser (Model ZEN3600, Malvern instruments Ltd., Malvern, UK).

### Transmission electron microscopy (TEM)

Samples for TEM analysis were prepared by drop-coating of GCNF at a concentration of 50 μg/ml on carbon-coated copper TEM grids. The grids were allowed to dry prior to measurement. TEM measurements were performed at an accelerating voltage of 120 kV on a Tecnai^TM^ G2 spirit (FEI Company, Eindhoven, Netherlands) instrument. Further, EDAX analysis was also employed for the elemental analysis of GCNF.

### Scanning electron microscopy (SEM)

SEM analysis was carried out for the surface morphological analysis of GCNF. Briefly, a very less amount of powder sample was put on carbon tape, sputter coated with gold and visualized at an accelerating voltage of 30 kV on a FEI Quanta FEG 450 field emission scanning electron microscope with EDAX (FEI Company, Eindhoven, Netherlands) instrument.

### X-ray photoelectron spectroscopy (XPS)

XPS measurements were performed for the measurement of carbon to oxygen ratio in GCNF (before and after sonication process) using PHI 5000 Versa Prob II, FEI Inc. spectrometer using nonmonochromatic Al Kα radiation (1486.6 eV). XPSPEAK41 software with a Gaussian−Lorentzian line shape was used for the deconvolution of individual spectral peaks.

### Cell culture and exposure to nanoparticles

The human lung adenocarcinoma (A549) cells were used in this study as in vitro models. A549 cells were procured from the American Type Culture Collection (ATCC, Manassas, VA) and cultured in DMEM F-12 culture medium (Life Technologies Pvt. Ltd., Invitrogen, Carlsbad, CA, USA) supplemented with 10% heat in-activated FBS, 0.2% sodium bicarbonate and 10 ml/L antibiotic antimycotic solution at 37 ^0^C under a humidified atmosphere of 5% CO_2_. Cell passage up to 10 was used for the experiments. At 80–90% confluency, cells were harvested using 0.25% trypsin - EDTA solution and were sub cultured into 96 well plate, 12 well plate, 6 well plate, and 75 cm^2^ culture flask according to the selection of experiment. Prior to treatment, cells were allowed to attach the culture surface for 22 h and then exposed to freshly prepare varying concentrations (1, 10, 25, 50 and 100 μg/ml) of GCNF (as prepared in preparation section) for different time point (1 – 48 h) according to the need of an assay. Each group contains four biological replicate and the treatment were performed for three times for each experiment. In each assay cells without nanoparticles were used as a control.

## Cellular internalization assessment of GCNF

### Flow cytometry based cellular uptake analysis

Flow cytometry based internalization assessment of GCNF in cultured cells was carried out according to the defined protocol [35]. According to this method, increase in the intensity of side scattered (SSC) light with constant intensity of forward scattered (FSC) light in exposed cells is the remark of cellular uptake of nanoparticles in the cells. In brief, 1 x 10^5^ cells/ml/well were seeded in 12 well culture plate and allowed to attach the surface for 22 h. Then cells were exposed to varying concentrations (1 – 100 μg/ml) of GCNF for 24 h and 48 h time period. After completion of exposure time, cells were washed with 1 X PBS to remove the excess GCNF and to avoid the possible interference with the experiment. Further, cells were harvested using 0.25% trypsin and centrifuged at 1000 rpm for 10 min. Supernatant was discarded and the pellet was re-suspended in 500 μl of 1 X PBS. Then analysis was made using flow cytometer equipped with 488 nm laser (FACS Canto^TM^ II, BD Biosciences, San Jose, CA, USA) instrument coupled with FACS Diva software (version 6.1.2, BD Biosciences, San Jose, CA, USA). Three independent experiments were performed for each group and values represents mean ± SE of three independent experiment. *p<0.05 was considered as statistical significant.

### Transmission electron microscopy analysis

Ultrathin section of cells exposed to GCNF were analysed using TEM for cellular internalization. Briefly, 2 x 10^5^ cells/ml/well were seeded in 6 well plate and exposed to 25 μg/ml concentration of GCNF for 24 h time period. After exposure, the treatment was aspirated and cells were washed with 1 X PBS to remove the excess NM present on the surface of cells. Further control and exposed cells were harvested using 0.25% trypsin and centrifuged at 1000 rpm for 10 min. Supernatant was discarded cells were fixed with 2.5% glutaraldehyde for 4 h followed by washing with 0.1 M sodium cacodylate buffer and post fixed in 1% osmium tetraoxide for 4 h at 4 ^0^C. Then fixed cells were washed with 0.1 M sodium cacodylate buffer, dehydrated through grades of acetones (15% – 100%) and infiltrated with Araldite^R^ – DDSA mixture for overnight at room temperature. The next day, samples were embedded in pure resin and blocks were cured at 60 ^0^C for 24 h. Then ultrathin sections (60 nm) of cells were cut using Leica UC7 ultra microtome (Wetzlar, Germany) and stained with Uranyl acetate and lead citrate. The stained sections were analyzed using Tecnai^TM^ G2 spirit (FEI Company, Eindhoven, Netherlands) instrument at an accelerating voltage of 80 kV equipped with Gatan camera.

### Internalization mechanism assessment

A549 cells were seeded in 12 well culture plate at a density of 1 x 10^5^ cells/ml/well and allowed to attach the surface for 22 h. Then cells were incubated with different inhibitors i.e. sodium azide (10 mM), amiloride (1 mM), wortmanin (23 μM), chlorpromazine (30 μM), sucrose (0.45 M) for additional 2 h. Then cells were exposed to 25 μg/ml concentration of GCNF for 24 h. For 4 ^0^C experiment, all aspects of experiment were carried out at 4 ^0^C instead of 37 ^0^C. After completion of exposure time, cells were washed with 1 X PBS to remove the excess GCNF and to avoid the possible interference with the experiment. Next, cells were harvested using 0.25% trypsin and centrifuged at 1000 rpm for 10 min. Supernatant was discarded and the pellet was re-suspended in 500 μl of 1 X PBS. Three independent experiments were performed for each group. Then analysis was made using flow cytometer equipped with 488 nm laser (FACS CantoTM II, BD Biosciences, San Jose, CA, USA) instrument coupled with FACS Diva software (version 6.1.2, BD Biosciences, San Jose, CA, USA). The dose of each inhibitor was chosen according to the previous studies [36–38]. Also, the effect of each inhibitor on the cell viability was determined using MTT assay. Values represents mean ± SE of three independent experiment. *p<0.05 was considered as statistical significant.

## Cytotoxicity assessment

### MTT assay

Viability of cells exposed to varying concentrations of GCNF was measured by MTT assay according to the defined protocol [39]. Briefly, cells (1 x 10^4^ cells/100 μl/well) were seeded on 96 well plates and exposed to 1 – 100 μg/ml of GCNF for 1 h to 48 h. Upon completion of exposure time, MTT solution (stock concentration: 5 mg/ml; working concentration: 10 μl/100 μl) was added to each well and then incubated for 3 h at 37 ^0^C. The resultant formazan crystals were solubilized with 200 μl of DMSO and the absorbance was quantified at 570 nm using the micro plate spectrophotometer system (SYNERGY-HT multiwell plate reader, Bio-Tek, Winooski, Vermont, USA) using KC-4 software. Three independent experiments were performed for each group. Along with all groups, cell free group (only GCNF and dye) was also included in the experiment to assess the possible interference of GCNF with MTT dye, if any present. The viability of the treatment group was expressed as a percentage of the control group, which was considered as 100%. Values represents mean ± SE of three independent experiment. *p<0.05 was considered as statistical significant.

### Propidium iodide (PI) dye exclusion assay

Membrane integrity analysis of A549 cells exposed to GCNF was carried out using flow cytometry based PI dye exclusion assay. Briefly, cells (1 × 10^5^ cells/ml/well) were seeded in 12 well culture plate and exposed to varying concentrations (1 – 100 μg/ml) of GCNF for 24 h and 48 h. After completion, cells were washed with 1 X PBS to remove the excess GCNF and to avoid the possible interference with the experiment. Further, cells were harvested and resuspended in 100 μl of 1 X PBS and incubated with PI dye (stock: 1 mg/ml; working: 2 μl/100 μl) for 10 min at room temperature. Next, dilution was carried out by adding 400 μl of 1 X PBS and red fluorescence emitted from PI was collected using BD FACS Canto II flow cytometer (BD Biosciences, San Jose, CA, USA) coupled with 650 ± 13 nm band pass filter. Three independent experiments were performed for each group. The proportion of cells with compromised membrane integrity was analyzed using FACS Diva software (version 6.1.2, BD Biosciences, San Jose, CA, USA). Values represents mean ± SE of three independent experiment. *p<0.05 was considered as statistical significant.

### Measurement of intracellular reactive oxygen species (ROS) generation

The intracellular ROS generation was measured by method of Wan et al. [40] and modified by Wilson et al. [41]. Briefly, cells (1 x 10^4^ cells/100 μl/well) were seeded in 96 well black bottom plate and exposed to varying concentrations (1 – 100 μg/ml) of GCNF for 1 h to 24 h. After completion of exposure time GCNF containing medium was aspirated and cells were washed twice with cold 1 X PBS to avoid the possible interference with the experiment. Thereafter, 100 μl of 1 X PBS containing DCFDA dye (20 μM) was added to each well. The plate was incubated for 30 min at 37 ^0^C and then DCFDA was discarded. Thereafter, 200 μl of 1 X PBS was added to each well and fluorescence intensity was measured in SYNERGY-HT multiwell plate reader (Bio-Tek, Winooski, Vermont, USA) using KC-4 software at excitation and emission wavelengths of 485 nm and 528 nm, respectively. Three independent experiments were performed for each group. Along with all groups, cell free group (only GCNF and dye) was also included in the experiment to assess the possible interference of GCNF with DCFDA dye, if any present. Values represents mean ± SE of three independent experiment. *p<0.05 was considered as statistical significant.

### Oxidative stress markers analysis

Cells were cultured in T - 75 cm^2^ culture flasks at a final density of ~6 x 10^6^ and exposed for 6 h with 1 – 100 μg/ml concentration of GCNF. After exposure, cells were washed twice with cold 1 X PBS and harvested in chilled 1 X PBS using cell scrapper. Then, cells were lysed using probe sonicator for 10 s total time and centrifuged at 1200 rpm for 10 min. The supernatant was discarded and the pellet was re-suspended in 1 X PBS to obtain cell lysate. Protein content was measured by Bradford method [42] using BSA as standard.

### Lipid peroxidation (LPO) measurement

The LPO measurement was carried out according to the method of Utley et al. [43] by estimating malondialdehyde (MDA) formed with 2-thiobarbituric acid (TBA). Three independent experiments were performed for each group. Values represents mean ± SE of three independent experiment. *p<0.05 was considered as statistical significant.

## Apoptosis analysis in GCNF exposed A549 cells

### Assessment of cell cycle progression of GCNF treated cells

Cells treated with different concentrations (1 – 100 μg/ml) of GCNF for 24 h were washed twice with cold 1 X PBS, harvested and centrifuged at 1200 rpm for 10 min and the pellet was resuspended in 300 μl of 1 X PBS. Cells were fixed with chilled 70% ice cold ethanol and incubated overnight at -20 ^0^C. Then, cells were centrifuged at 1200 rpm for 4 min and pellet was again resuspended in 200 μl of lysis buffer (1 X PBS along with 0.2% Triton X-100) and incubated at 4 ^0^C for 30 min. Lysed cells were centrifuged at 1200 rpm for 10 min and pellet was resuspended in 1 ml of 1 X PBS containing 20 μl of RNase (10 mg/ml) and incubated for 30 min at 37 ^0^C. Cells were again centrifuged at 1200 rpm for 10 min and pellet was resuspended in 500 μl of 1 X PBS containing 10 μl of PI dye (1 mg/ml) and stored at 4 ^0^C until read at flow cytometer (FACS Canto^TM^ II, BD BioSciences, San Jose, CA, USA). Three independent experiments were performed for each group. Values represents mean ± SE of three independent experiment. *p<0.05 was considered as statistical significant.

### Determination of mode of cell death (apoptosis/necrosis)

Apoptosis kit (FITC Annexin V Apoptosis detection Kit, BD Biosciences, San Jose, CA, USA) was employed to detect apoptotic and necrotic cells after exposure of GCNF. The manual of the kit was strictly followed. Briefly, 1 x 10^5^ cells/ml/well were plated in the 12 well culture plate and incubated with different concentrations (1 – 100 μg/ml) of GCNF for 24 h. Upon completion of exposure time, cells were harvested, washed twice with cold 1 X PBS and re-suspended in 0.1 ml of 1 X binding buffer (supplied with FITC Annexin V Apoptosis detection Kit). Then cells were incubated with 5 μl of FITC-Annexin V and PI for 10 min at room temperature in dark. After incubation, 0.4 ml of 1 X binding buffer was further added to each sample and cells were analyzed using flow cytometer (FACS Canto^TM^ II, BD BioSciences, San Jose, CA, USA). Three independent experiments were performed for each group. Values represents mean ± SE of three independent experiment. *p<0.05 was considered as statistical significant.

### Mitochondrial membrane potential (MMP) analysis using lipophilic fluorochrome

For the MMP analysis, fluorescence intensity of lipophilic cationic JC - 1 dye was used as a reporter. This dye has dual fluorescence nature and upon the induction of loss in MMP, fluorescence of this dye changes from red to green.

Briefly, cells exposed to 1 – 100 μg/ml concentrations of GCNF for 24 h were washed with 1 X PBS and incubated with 10 μM JC - 1 dye in culture medium for 15 min at 37 ^0^C. Then cells were again washed with and re-suspended in 400 μl of 1 X PBS. The cells were analyzed for red and green fluorescence in a BD FACS Canto II flow cytometer (BD Biosciences, San Jose, CA, USA) coupled with 485 nm wavelength excitation filter and 590 nm wavelength emission filter. Three independent experiments were performed for each group. Values represents mean ± SE of three independent experiment. *p<0.05 was considered as statistical significant.

## Autophagy analysis

### Monodansylcadaverine (MDC) Staining

MDC staining was introduced to analyze autophagy induction in cells. After exposure with 25 μg/ml concentration of GCNF, cells were rinsed with 1 X PBS and stained with 50 mM MDC at 37 ^0^C for 1 h. Finally, cells were washed with 1 X PBS, and the cellular fluorescence changes were observed using Nikon Eclipse Ti-S inverted fluorescent microscope equipped with Nikon Digital slight Ds-Ri1 CCD camera and NIS element BR imaging software (Nikon, Minato Tokyo, Japan). Three independent experiments were performed for each group and the representative image has been showed in the result.

### Transmission electron microscopy

For TEM analysis, A549 cells were exposed to 25 μg/ml concentration of GCNF for 24 h. Then cells were washed with 1 X PBS, to remove the excess nanomaterials present on the surface of cells followed by harvesting using 0.25% trypsin and centrifuged at 1000 rpm for 10 min. Further, cells were fixed with 2.5% glutaraldehyde for 4 h followed by washing with 0.1 M sodium cacodylate buffer and post fixed in 1% osmium tetraoxide for 4 h at 4 ^0^C. Then fixed cells were washed with 0.1 M sodium cacodylate buffer, dehydrated through grades of acetones (15 – 100%) and infiltrated with Araldite^R^ – DDSA mixture for overnight at room temperature. The next day, samples were embedded in pure resin and blocks were cured at 60 ^0^C for 24 h. Then ultrathin sections (60 nm) of cells were cut using Leica UC7 ultra microtome (Wetzlar, Germany) and stained with Uranyl acetate and lead citrate. The stained sections were analyzed using Tecnai^TM^ G2 spirit (FEI Company, Eindhoven, Netherlands) instrument at an accelerating voltage of 80 kV equipped with Gatan camera.

### Immunoblotting

Upon the completion of indicated exposure time, cells were washed twice with cold 1 X PBS, harvested and the whole-cell extracts was prepared using cell lytic reagent (Sigma-Aldrich, St. Louis, Missouri, USA) supplemented with protease and phosphatase inhibitors. Then protein samples were resolved by SDS–polyacrylamide gel electrophoresis and transferred to PVDF membrane by electro blotting. The membrane was blocked with casein blocking buffer (Sigma-Aldrich, St. Louis, Missouri, USA) for 1 h at room temperature and incubated with primary antibodies in 1 X TBST for overnight at 4 ^0^C. After washing with 1 X TBST, the blots were further incubated with the corresponding horseradish peroxidase-coupled anti-rabbit or anti-mouse secondary antibody (Cell Signaling Technology Inc., Danvers, MA, USA). Proteins were visualized with Super Signal West Femto reagents (Pierce Biotechnology, Rockford, IL) and chemiluminiscence was detected. Three independent experiments were performed for each group. Values represents mean ± SE of three independent experiment. *p<0.05 was considered as statistical significant. Levels of GAPDH signal were used to verify equal protein loading in each lane.

### Plasmid and siRNA transfection in A549 cells

Prior to GCNF exposure, A549 cells were transiently co-transfected with plasmid for mammalian GFP - LC3, Mito - DsRed and LC3 - siRNA. Briefly, cells were plated in the 4 well chamber slides and conventional lipid mediated gene delivery using lipofectamine 2000 for plasmid and lipofectamine RNAiMax for siRNA (Invitrogen, Carlsbad, CA, USA) was performed according to the manufacturer’s instruction. Following transfection, cells were exposed to 25 μg/ml concentrations of GCNF for 24 h. Then cells were washed with 1 X PBS and fixed in 4% paraformaldehyde and mounted using antifade for microscopic analysis or subjected to protein isolation.

### Quantification of GFP - LC3 puncta

For the counting of GCNF induced GFP - LC3 puncta, 50 cells from each group was blindly selected by a person who was unknown to experimental design. In each cell, puncta was counted, averaged according to the well established method [44] and results were expressed as mean ± SE of three independent experiments. *p<0.05 was considered as statistical significant.

## Lysosomal membrane permeabilization analysis

### Acridine orange staining

Acridine orange staining of A549 cells exposed with GCNF was carried out to assess the lysosomal membrane permeabilization. Briefly, 1 X 10^5^ cells/ml/well were seeded in 12 well plate, exposed to GCNF (1 – 100 μg/ml concentration), washed twice with 1 X PBS, harvested and resuspended in 1 X PBS. Following stained with 10 μg/ml solution of acridine orange dye for 10 min, the cells were analyzed for decrease in red fluorescence using BD FACS Canto II flow cytometer (BD Biosciences, San Jose, CA, USA) coupled with 485 nm wavelength excitation filter and 590 nm wavelength emission filter. Three independent experiments were performed for each group.

### Immunofluorescence study

A549 cells were plated onto 4 well chamber slides and exposed to 25 μg/ml concentration of GCNF for 24 h. Following, cells were washed with 1 X PBS, stained with Lysotracker Red dye (working concentration – 50 nM, Incubation – 30 min at 37 ^0^C), pHrodo green dextran (working concentration – 25 μg/ml, Incubation – 15 min at 37 ^0^C), Oregon green 488 Phalloidin (working concentration – 0.5 μM, Incubation – 20 min at room temperature), and incubated with primary p62 antibody (working dilution – 1 : 200, Incubation – overnight at 4 ^0^C) followed by secondary antibody. The cells were mounted and examined under a Nikon Eclipse 80i upright fluorescent microscope equipped with Nikon Digital slight Ds-Ri1 CCD camera and NIS element BR imaging software (Nikon, Minato Tokyo, Japan). Three independent experiments were performed for each group. Values represents mean ± SE of three independent experiment. *p<0.05 was considered as statistical significant.

## Energetic impairment analysis

### ATP measurement assay

Intracellular ATP level of GCNF exposed (1 – 100 μg/ml concentration), A549 cells were measured using ATP assay kit from Abcam, Cambridge, UK as per manufacture’s instruction. Three independent experiments were performed for each group. Values represents mean ± SE of three independent experiment. *p<0.05 was considered as statistical significant.

### Glucose uptake assay

Glucose uptake analysis of GCNF exposed cells (1 – 100 μg/ml concentration) were carried by measuring fluorescence of 2 – NBDG (Sigma-Aldrich, St. Louis, Missouri, USA) using BD FACS Canto II flow cytometer (BD Biosciences, San Jose, CA, USA) coupled with 485 nm wavelength excitation filter and 590 nm wavelength emission filter. Three independent experiments were performed for each group. Values represents mean ± SE of three independent experiment. *p<0.05 was considered as statistical significant.

### Quantitative real time PCR

Total RNA from control A549 cells and GCNF exposed cells at a concentration of 25 μg/ml was extracted using Trizol reagent (Thermo Fisher Scientific, Waltham, Massachusetts, USA) according to the manufacturer’s instruction. Following, cDNA was prepared using high capacity reverse transcription kit (Thermo Fisher Scientific, Waltham, Massachusetts, USA) and altered gene expression level compared to control was assessed by quantitative real time RT-PCR with SYBR Green as a fluorescent reporter using Maxima SYBR Green/ROX PCR Kit (Thermo Fisher Scientific, Waltham, Massachusetts, USA) and QuantStudio^TM^ 6 Flex Real Time PCR System (Thermo Fisher Scientific, Waltham, Massachusetts, USA). GAPDH and 18S RNA was served as internal control. Calculation for relative gene expression was carried out according to the DDCt method using Threshold values. Three independent experiments were performed for each group. Values represents mean ± SE of three independent experiment. *p<0.05 was considered as statistical significant.

### Assessment of DNA damage Induction by GCNF in A549 cells

DNA damaging potential of GCNF was assessed using Comet assay (standard alkaline and Fpg modified) and flow cytometry based micronucleus formation (MN) assay.

### Standard alkaline comet assay/single cell gel electrophoresis

The induction of damage to deoxyribonucleic acid (DNA) by NM in cultured cells was assessed by using alkaline Comet assay according to the method of Singh et al. [45] and base slides were prepared according to the method of Bajpayee et al. [46].

Briefly, 1 x 10^5^ cells/ml/well seeded in 12 well culture plate was exposed to different concentrations (1 – 100 μg/ml concentration) of GCNF for 6 h. Following the exposure, cells were washed twice with 1 X PBS to avoid interference, harvested using trypsin-EDTA solution, and re-suspended in 100 μl of 1 X PBS. Thereafter, the cell suspension was mixed with 1% low melting point agarose (LMPA, prepared in 1 X PBS) to achieve a final concentration of 0.5%. Then 80 μl of the suspensions were layered on base slide (pre coated with 1% NMA), evenly spread with cover slip and kept on ice to allow the gel to solidify. The cover slip was removed carefully and third layer of 90 μl of 0.5% LMPA was added. Again the layer was evenly spread with cover slip and kept on ice to allow the gel to solidify. Duplicate slide for each sample was prepared.

As prepared slides were kept in freshly prepared and chilled lysis solution [146.1 g sodium chloride (NaCl), 37.2 g EDTA, 1.2 g Tris, pH 10 with 1% Triton X 100 added just before use] at 4 ^0^C for overnight. After lysis, the slides were placed in a horizontal gel electrophoresis tank containing freshly prepared chilled electrophoresis solution (1 mM EDTA, 300 mM NaOH, pH > 13) for 20 min for DNA unwinding and subsequently subjected to electrophoresis with 300 mA current, 0.7 V/cm at 4 ^0^C under dimmed light for 30 min.

After electrophoresis, the slides were neutralized for excess alkali solution with Tris buffer (0.4 M, pH 7.5) for 3 times of 5 min cycle and stained with 75 μl of ethidium bromide (EtBr) solution (20 μg/ml). Slides were stored in a humidified slide box until score. The scoring of the slides was done at X 400 magnification using fluorescent microscope (DMLB, Leica, Germany) coupled with CCD camera and image analysis system (Andor Technology, Belfast, UK). The analysis was done using image analysis software (KOMET 5.0, Kinetic Imaging, U.K.) attached with microscope. Three independent experiments were performed for each group. As per the Comet assay guideline [47, 48], 50 cells for per group were scored. The mean value of three Comet parameters tail DNA (%), tail length (μm) and Olive tail moment (OTM) was considered during the expression of result. Values represents mean ± SE of three independent experiment. *p<0.05 was considered as statistical significant.

### Fpg modified comet assay

To determine the role of oxidative stress in NM induced DNA damage, the Fpg modified Comet assay was performed according to the defined protocol of Tice et al. [48]. The process up to the lysis step is similar to the alkaline Comet assay. After lysis, slides were washed three times in enzyme buffer (40 mM HEPES, 0.1 M KCl, 0.5 mM EDTA, pH 8, 0.2 mg/ml BSA) and incubated with 1 : 3000 Fpg enzyme solution for 30 min at 37 ^0^C. Further, the slides were processed as in the alkaline Comet assay. Three independent experiments were performed for each group. Values represents mean ± SE of three independent experiment. *p<0.05 was considered as statistical significant.

### Flow cytometry based micronucleus (MN) assay

This assay was used to assess the cytogenetic damage caused by the GCNF that result into the formation of micronuclei (MN). The method was carried out according to the protocol of Nüsse and Marx [49] and modified by Pandey et al. [50].

Briefly, 2 x 10^5^ cells/ml/well were seeded in 6 well culture plate and exposed to different concentrations (1 – 100 μg/ml) of GCNF for 3 h and 6 h. After completion of exposure time, the treatment was aspirated and cells were washed with serum free culture medium and further cultured for 24 h in culture medium supplemented with 10% foetal bovine serum to complete one more cell division cycle. Further, cells were washed twice with 1 X PBS to avoid interference, harvested using 0.25% trypsin-EDTA solution and centrifuged at 250 x g for 10 min at room temperature. The supernatant was discarded and the cell pellet was resuspended by vortexing in 1 ml of solution I containing 10 mM NaCl, 3.4 mM sodium citrate, 10 mg/L RNase, 0.3 mg/L IGEPAL, 25 mg/L EtBr and incubated at room temperature for 1 h. Then, to this 1 ml of solution II containing 1.5% citric acid, 0.25 M sucrose, 40 mg/L EtBr was added and the solution mixture was incubated for another 15 min at room temperature. After the completion of incubation, the samples were analyzed using BD FACS Canto II flow cytometer coupled with FACS Diva software (version 6.1.2, BD Biosciences, San Jose, CA, USA). Three independent experiments were performed for each group. Values represents mean ± SE of three independent experiment. *p<0.05 was considered as statistical significant.

### Statistical analysis

Results were expressed as mean ± SE of three experiments and data were analyzed using Graphpad InStat statistical software (SanDiego, CA, USA). Mean significant difference between experimental groups was determined using one way analysis of variance (ANOVA) with Tukey-Kramer post hoc multi comparison test to determine significance. Homogeneity of variance between all groups was ascertained. In all cases, *p* < 0.05 was considered as statistical significant.

## Results

### Characterization, internalization of GCNF and ultra structural changes in A549 cells

**Fig. 1:**
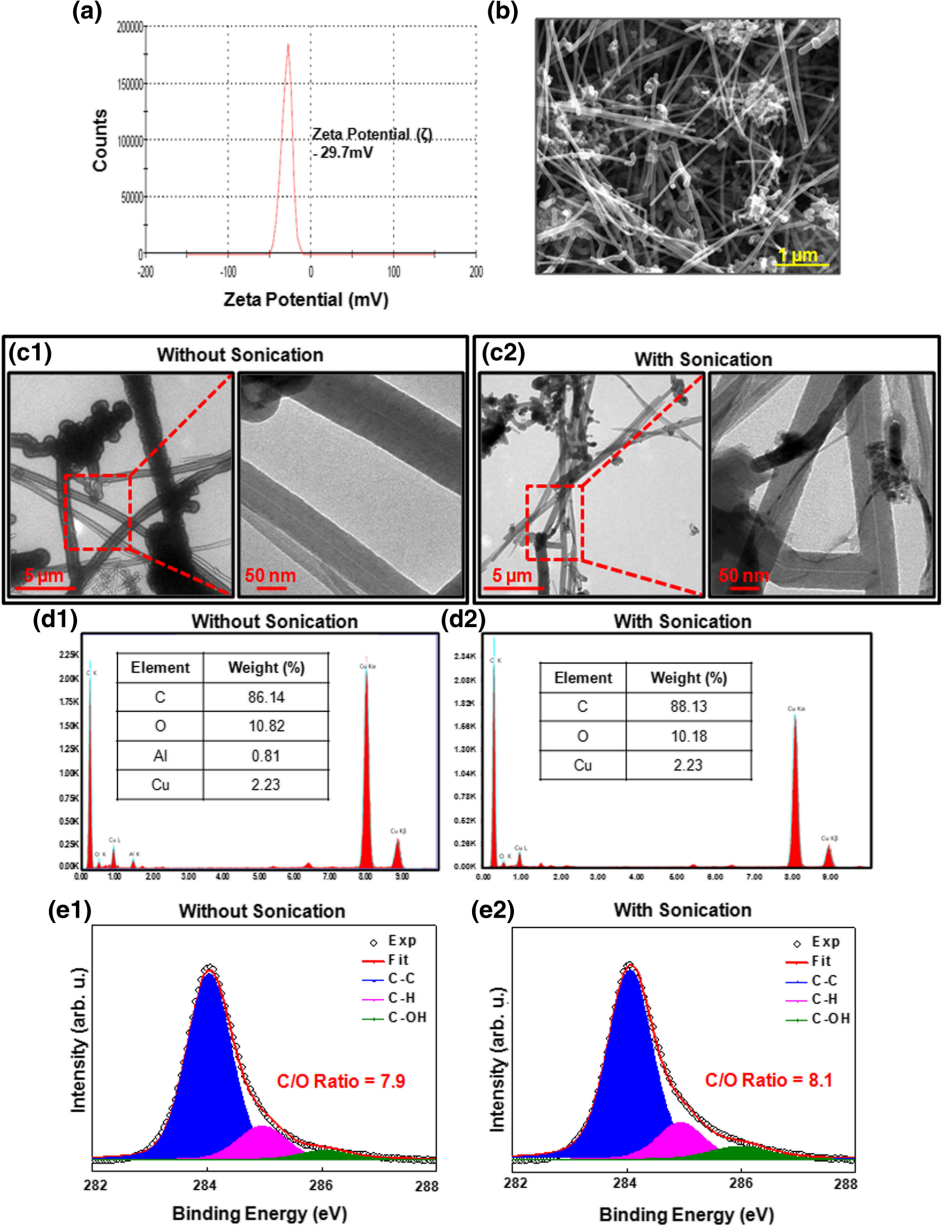
Characterization of GCNF using (**a**) Dynamic light scattering (DLS) for zeta potential measurement, (**b**) Scanning electron microscopy for shape. (**c**) Transmission electron microscopy analysis of GCNF in, without sonication (c1) and after sonication (c2) condition showed that sonication does not affect the morphology of GCNF. (**d**1, **d**2) EDAX analysis was used for the elemental analysis which showed that GCNF contains very negligible amount of impurities. (**e**1, **e**2) X-ray photoelectron spectroscopy further showed that upon sonication there was no effect in the carbon to oxygen ratio of GCNF

Since the physico-chemical properties of NM can affect their biological/toxicological response thus the characterization of these properties is of prime importance during their toxicity evaluation [51]. In the present study, GCNF was characterized in culture medium, water as well as in dry state by employing DLS, TEM and SEM analysis, respectively. DLS analysis revealed that the zeta potential of GCNF in culture medium was found to be -29.7 mV (Fig. 1a). Zeta potential provides quantitative information about the stability of NM in liquid with having value close to ± 30 mV zeta potential showed more stability [52]. Further, stability of GCNF dispersion was assessed using DLS based on zeta potential measurement. In our study, GCNF also showed the zeta potential close to the stability value over the time period of 48 h ensured their good dispersion in culture medium during exposure (Table 1). SEM analysis revealed typical fibrous shape of GCNF (Fig. 1b). Further, TEM analysis clearly demonstrated that majority of GCNF were in the range of outer diameter of 79 ± 6.6 nm along with inner diameter of 7 ± 0.8 nm (Fig. 1c1, c2). However, some of the nanoparticles were also in the range of 110 ± 10 nm of outer diameter which showed the polydisperse nature of GCNF in the sample. Further, length measurement using TEM analysis showed that the GCNF were having a length of 25 ± 10 μm (Fig. 1c1, c2). This configuration of GCNF was comparable to the standard respirable particle size limit (i.e. ˂ 3 μm). Next, to assess the impact of probe sonication on GCNF morphology, we further characterize our GCNF prior and after sonication using TEM analysis for their diameter and length measurement. Results showed that the sonication process had very little or negligible effect on the GCNF properties as shown in Fig. 1c1, c2. Further, we carried out the elemental analysis of GCNF sample using EDAX analysis and found there were very less metal impurities was present as shown in Fig. 1d1, d2 These observations suggested that GCNF are of fibrous shape NM with respirable size limit in the nanoscale range which can pose threat to lung cells and tissue after their internalization through inhalation.

**Table 1:** Zeta potential measurement for the assessment of solution stability using dynamic light scattering

Time (h)	Zeta potential (mV)
0	-29.3
1	-29.5
3	-28.7
6	-28.2
24	-27.6
48	-26.1

**Fig. 2:**
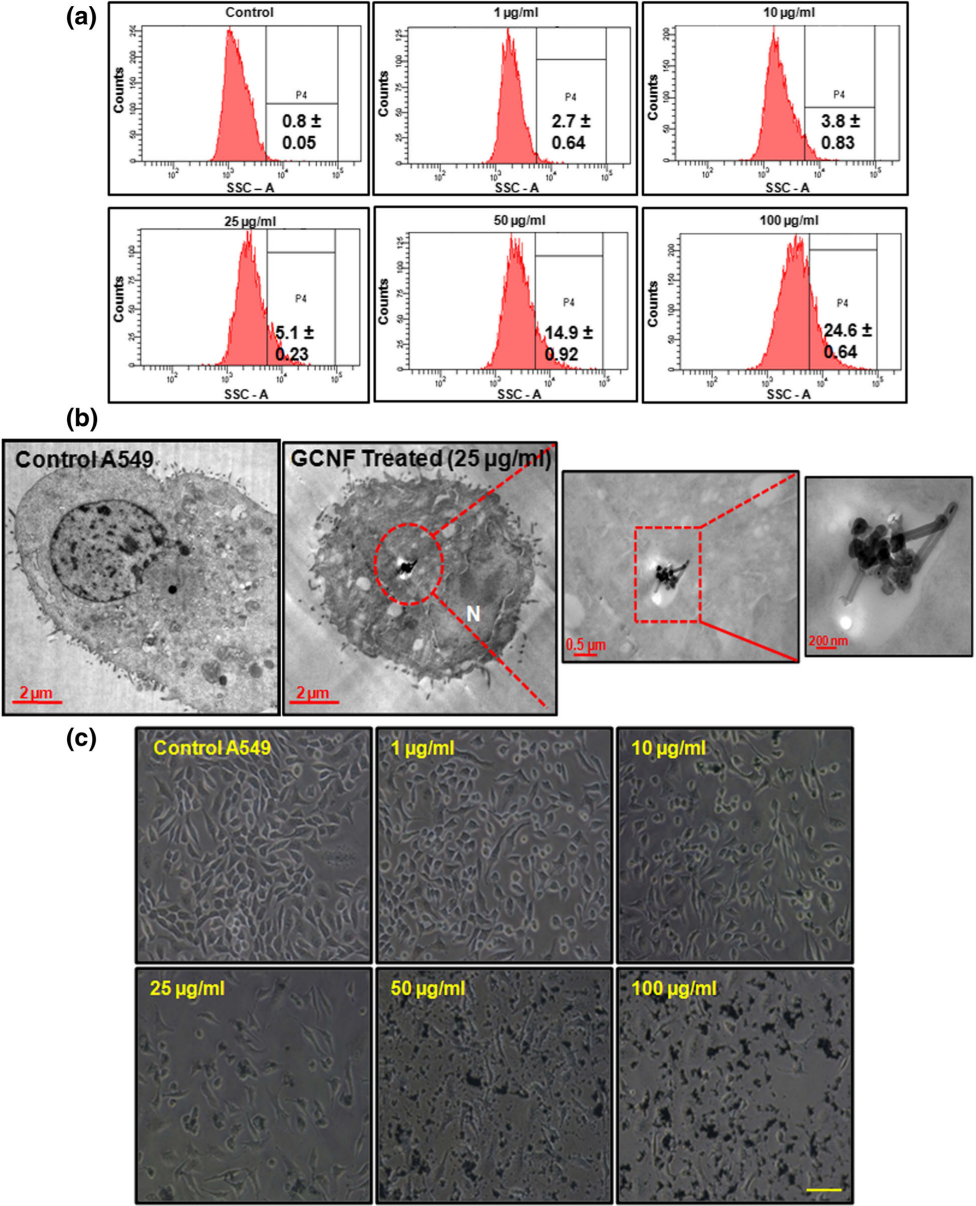
Representative flow cytograms (**a**) and corresponding TEM images (**b**) depicting cellular internalization of GCNF in A549 cells. In TEM images, red enclosure denotes presence of GCNF in membranous structure. (**c**) Phase contrast photomicrographs of A549 cells showing their cellular morphology. For flow cytometry, three independent experiments were performed and data represents mean ± SE value. *p<0.05 was considered as statistical significant. Scale Bar – 300 μm

The XPS C1s core spectra were recorded for each sample which showed a significant comparable ratio between carbon to oxygen in both situations i.e. before and after sonication process. These results showed that the sonication had no effect on the oxidation of GCNF along with minimal effect on morphology (Fig. 1e1, e2). In order to characterize the interaction between GCNF and cells, we analyzed their internalization in A549 cells using flow cytometry and TEM analysis. A549 cells were exposed to varying concentrations (1 - 100 μg/ml) of GCNF for 24 h. Flow cytometry analysis indicated a significant increase (*p < 0.05) of 5.1%, 14.9%, 24.6% in SSC intensity, which is a marker of increased cell granularity, in GCNF treated A549 cells at 25 μg/ml, 50 μg/ml and 100 μg/ml respectively as compared to the untreated control (Fig. 2a).

Further, these observations were corroborated by TEM analysis (Fig. 2b) which showed the accumulation of electron dense GCNF in the cytoplasm as well as enclosed in vesicle in A549 cells. Next, the uptake mechanism analysis of GCNF in A549 cells showed their internalization facilitated by the receptor mediated endocytosis and moreover by the clathrin mediated process (Additional file 1: Figure S1). Including, not significant reduction in the viability of A549 cells, incubated with inhibitors, was found (data not shown). This accumulation was further found to cause many ultra-structural changes in the cells such as increased formation of vacuoles, elongation and swelling of mitochondria, dilation of endoplasmic reticulum (ER), condensation of chromatin as shown in further experiments. Including this, most cells showed diffuse cytoplasmic expansion and lost their adherence property in a concentration dependent manner as revealed by the morphological analysis (Fig. 2c).

### Effect of GCNF exposure on A549 cells viability

**Fig. 3:**
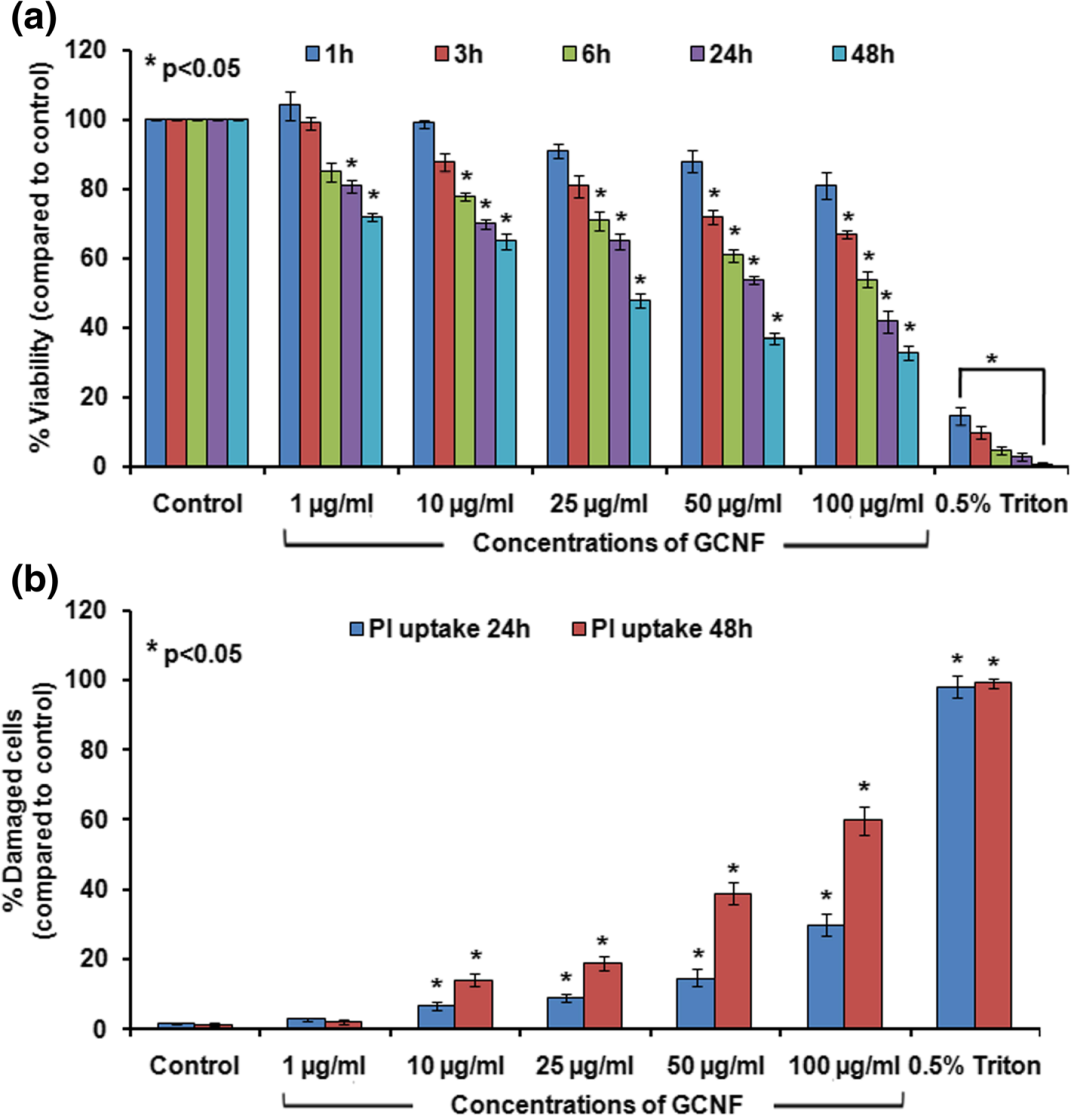
Viability reduction and loss of membrane integrity in GCNF exposed A549 cells was assessed using MTT assay (**a**) and propidium iodide (PI) dye exclusion assay (**b**), respectively. Values represents mean ± SE of three independent experiment. *p<0.05 was considered as statistical significant

The viability of GCNF treated A549 cells were studied by using MTT assay. A statistically significant decrease in viability (22%, 29%, 39% and 46% at 10 μg/ml, 25 μg/ml, 50 μg/ml and 100 μg/ml respectively) of A549 cells was noted as early as 6 h post treatment. This decrease in the viability was further enhanced at 24 h and reached to 35%, 52%, 63% and 67% at 10 μg/ml, 25 μg/ml, 50 μg/ml and 100 μg/ml after 48 h post treatment (Fig. 3a). Further, the membrane integrity analysis by PI dye exclusion assay showed a significant concentration and time dependent increase in the number of cells with compromised membrane after GCNF exposure (Fig. 3b). These results may be correlated with the above results showing the dose dependent internalization resulting in loss of membrane integrity and cell death after GCNF exposure.

### Autophagosomes accumulation, blockade of autophagic flux and mTOR pathway induction in GCNF exposed A549 cells

**Fig. 4:**
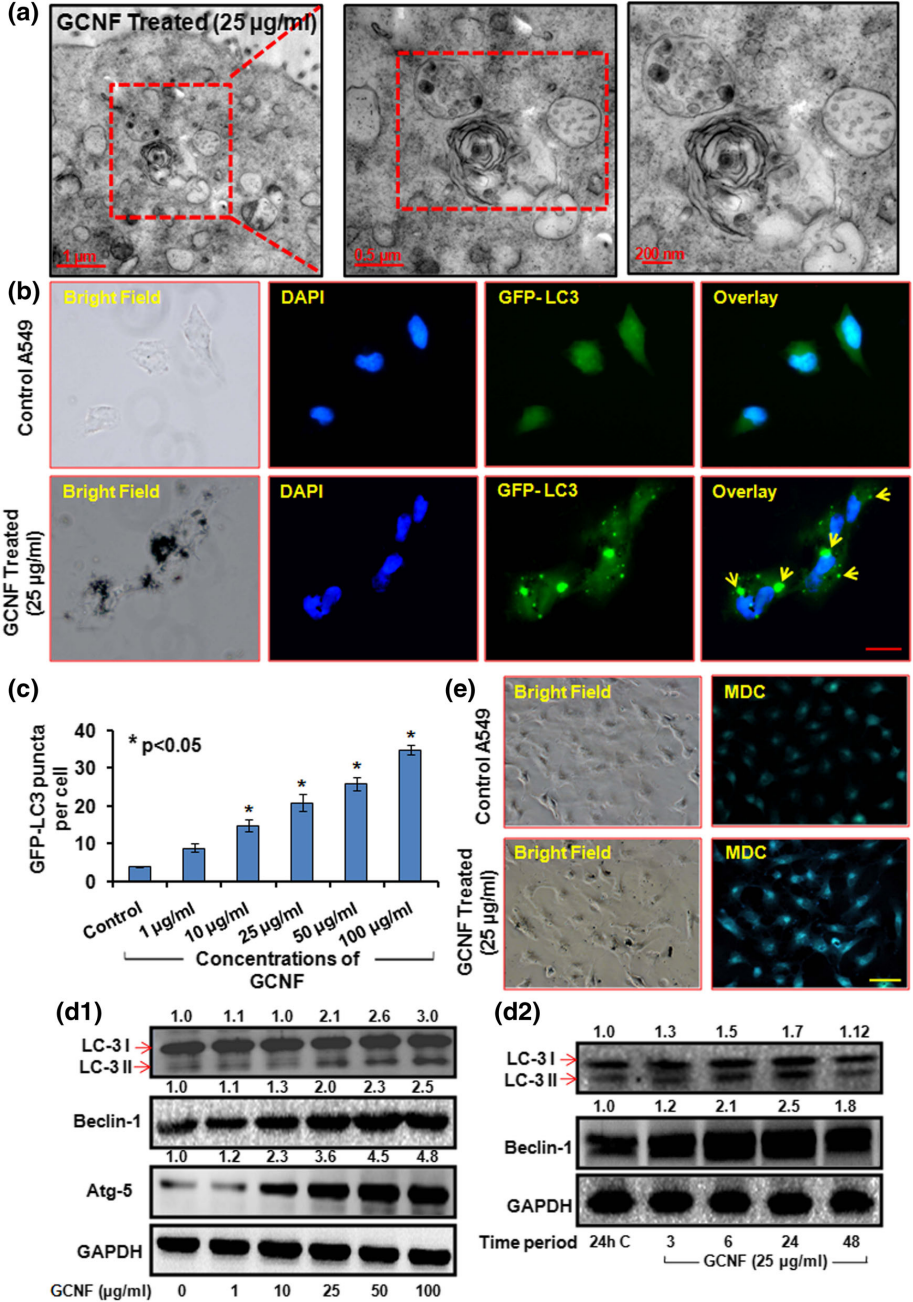
Representative TEM photomicrographs (**a**) and corresponding fluorescence microscopic images (**b**) portraying the autophagosomes accumulation in GCNF (25 μg/ml) exposed cells. In TEM image, inset of the red marked area present detailed structure of autophagosomes whereas in fluorescence images yellow arrows marked the GFP-LC3 puncta. Scale Bar – 20 μm. Per view 5 cells and 10 views per group were analyzed. (**c**) Quantification of autophagosomes per cell in fluorescence images. Values are expressed as mean ± SE of three independent experiment. *p<0.05 was considered as statistical significant. (**d** –1, 2) Effect of GCNF on autophagic proteins in dose dependent and time dependent manner analyzed through western blot. GAPDH was served as a loading control. Densitometry analysis is represented by the digits given above the respective blots and also provided in Additional file 2: Figure S2a, b. Values are expressed as mean ± SE of three independent experiment. *p<0.05 was considered as statistical significant. (**e**) Monodansyl cadaverine (MDC) staining of GCNF exposed cell. Scale Bar - 50 μm

Since, in our study GCNF was found to be accumulating in vesicles along with cellular materials which led us to investigate the effect of GCNF on autophagy in exposed A549 cells. In order to determine this, the formation of “double membrane autophagic vacuole - autophagosomes” – maker of autophagy, through TEM examination in GCNF exposed A549 cells was analyzed. TEM analysis clearly revealed the accumulation of autophagosomes in GCNF exposed cells compared to control cells (Fig. 4a).

Further autophagosomes accumulation was confirmed by observing the accumulation of green fluorescence protein (GFP) – tagged LC3 (GFP - LC3) puncta structures which was quite evident in GCNF exposed A549 cells (Fig. 4b, c) compared to control cells. Including this, accumulation of monodansylcadaverine (MDC) positive autophagic vacuoles in A549 cells following 24 h exposure of GCNF further corroborated these findings (Fig. 4e). The mRNA and protein expression level analysis of various autophagy markers such as LC3 – I/II, ATG5 and Beclin – 1 using Real Time PCR and western blot analysis, respectively depict an increased expression level of respective genes at both level (Fig. 4d1, d2 and Additional file 2: Figure S2). Thus the above results conclusively showed the accumulation of autophagosomes and indicate the possible role of autophagy in GCNF induced toxicity.

**Fig. 5:**
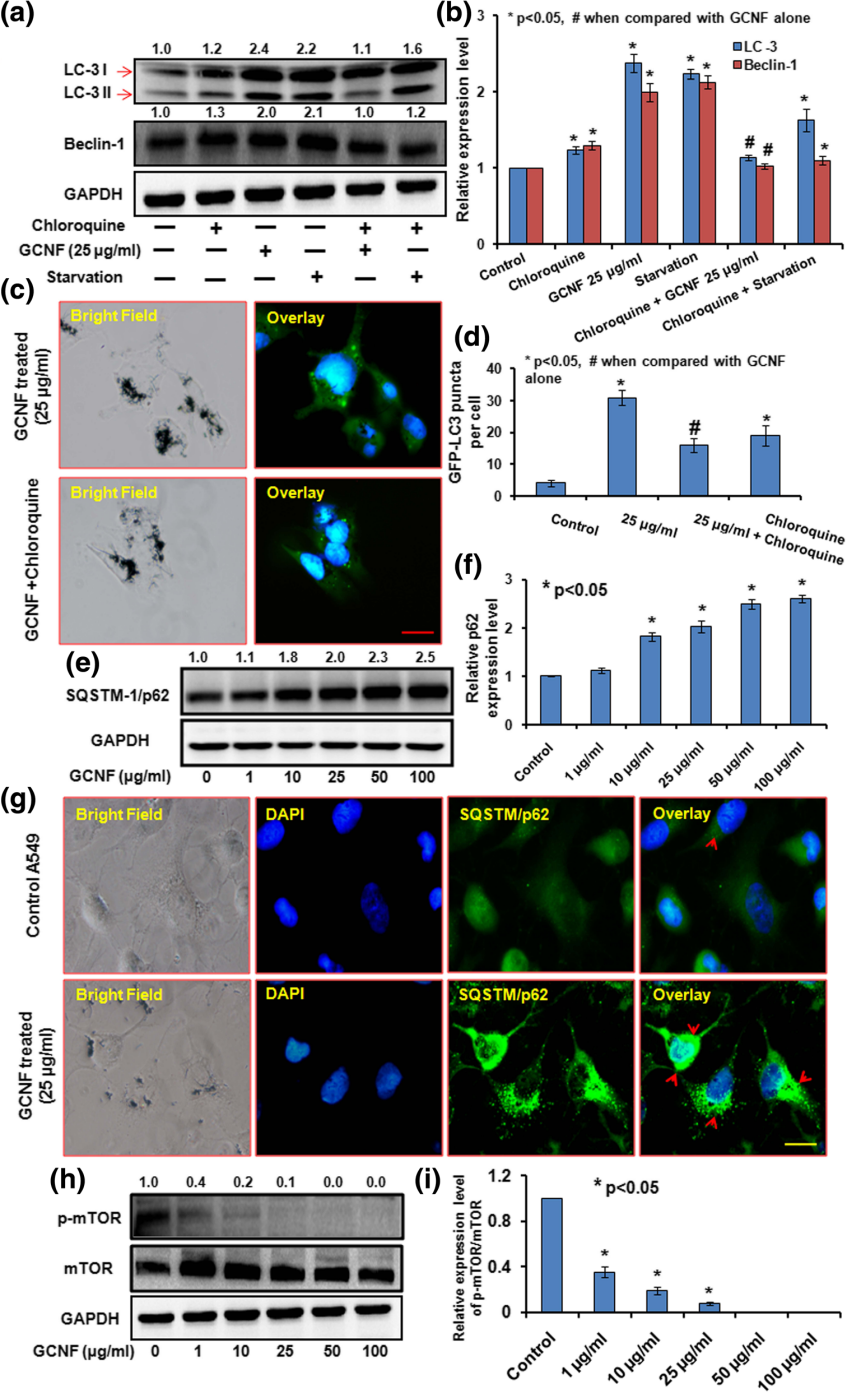
(**a**) Western blot analysis of LC3, Beclin-1 and (**b**) Respective densitometry analysis and (**c**) Representative fluorescence photomicrographs of GFP – LC3 plasmid transfected A549 and their respective statistical analysis (**d**) in the presence and absence of chloroquine to determine the autophagic flux. Scale Bar - 20 μm. Immunoblotting analysis (**e**), respective densitometry (**f**) and corresponding fluorescence photomicrographs (**g**) depicting the accumulation of SQSTM1/p62 after GCNF exposure in A549 cells. Scale Bar - 20 μm. Per view 10 cells and 4 views per group were analyzed. (**h **) Immunoblotting and (**i**) respective densitometry analysis of GCNF exposed A549 cells to assess the effect on mTOR signaling pathways. GAPDH was used as a loading control. Values are expressed as mean ± SE of three independent experiment. *p<0.05 was considered as statistical significant

Since, accumulation of autophagosomes can result either from enhanced induction of autophagy or by the blockade of autophagic flux [53]. Thus the autophagic flux assay was carried out to determine the actual scenario in GCNF exposed A549 cells. For this, we exposed A549 cells with chloroquine, a potent lysosomal activity inhibitor, prior to GCNF exposure and LC3 - II level with GFP - LC3 puncta index was studied. It was found that GCNF did not significantly alter the LC3 - II level along with GFP - LC3 puncta index in presence of chloroquine (Fig. 5a, b, c, d) which indicates that GCNF do not actually induce autophagy rather than possible blockade of autophagic flux.

Further western blot and florescence microscopic analysis of SQSTM - 1/p62 protein, that is preferentially degraded through autolysosomes, showed an accumulation of p62 in A549 cells (Fig. 5e, f, g) after exposure to GCNF. These results conclude an impaired autophagic flux in A549 cells during the exposure of GCNF causing the accumulation of autophagosomes.

Various signaling pathways have been shown to regulate autophagy, out of them PI3K/Akt/mTOR pathway is a classical pathway in which mTOR (mammalian target of rapamycin) gets inhibited [54]. Along this, it has also been reported that autophagy regulation could be an mTOR independent process along with the role of mTOR inhibition in autophagic flux disruption rather than autophagy induction [55–60]. In our study, GCNF exposure resulted in a significant dose dependent decrease in the level of phosphor-mTOR while the basal mTOR was not found to alter significantly (Fig. 5h, i). Thus, our results suggest that the inhibition of mTOR pathways leads to the accumulation of autophagosomes mediated through blockade of autophagic flux, rather than activation of autophagy.

### Lysosomal destabilization and cytoskeleton disruption mediated impaired fusion of autophagosomes

**Fig. 6:**
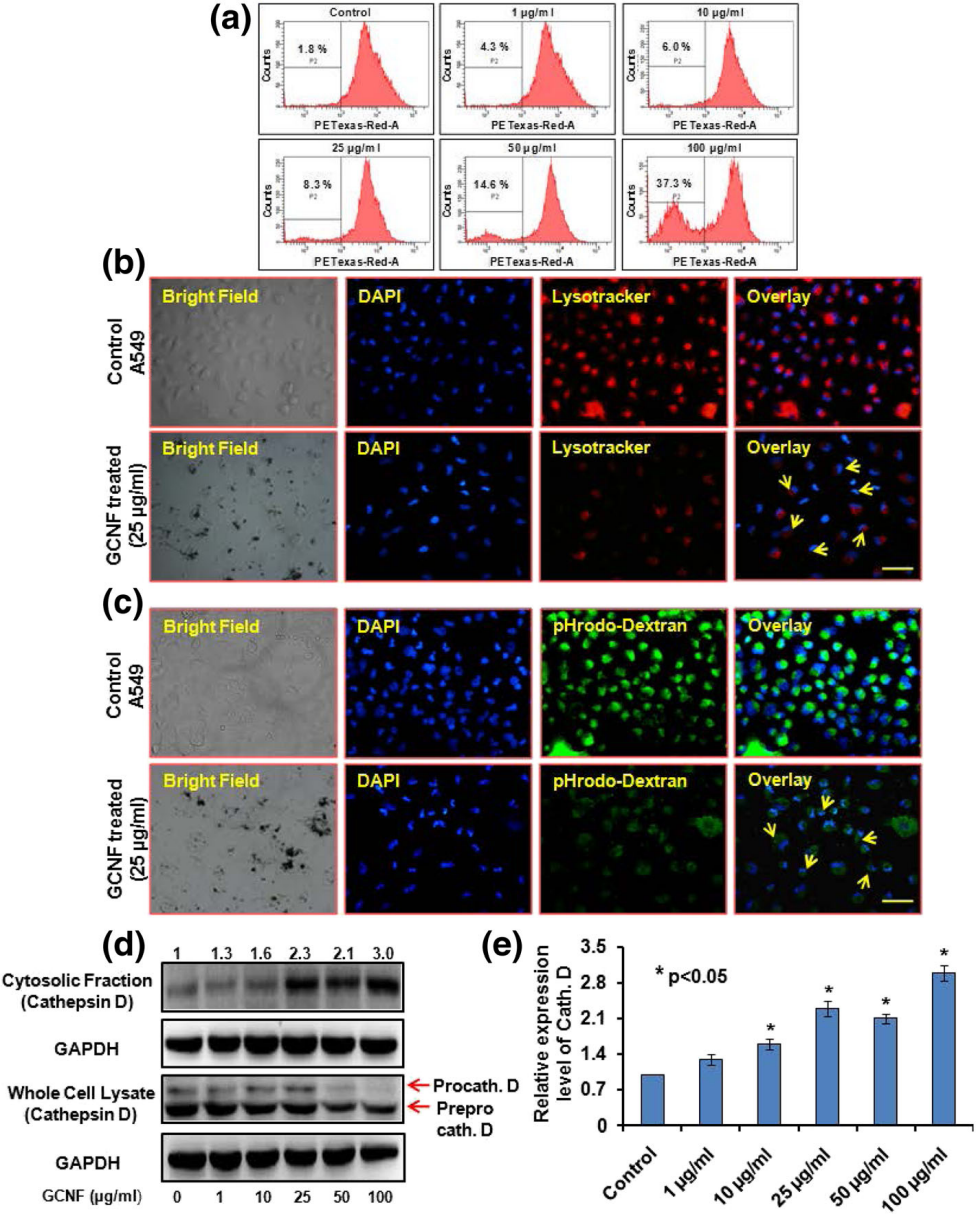
Representative flow cytograms and corresponding fluorescence photomicrographs showing (**a**) % reduction in acridine orange positive cells (**b**) reduced Lysotracker red staining (**c**) reduction in pHrodo dextran green fluorescence (marked with yellow arrows). Scale bar - 50 μm. Per view 50 cells and 4 views per group were analyzed. (**d**) Immunoblotting and (**e**) respective densitometry analysis of cytosolic release of cathepsin D in GCNF exposed cells. GAPDH was used as a loading control. Values are expressed as mean ± SE of three independent experiment. *p<0.05 was considered as statistical significant

Maturation of autophagosomes through its fusion with lysosomes, which forms autolysosomes, ensures proper disposal of damaged cellular biomolecules or organelles. Any perturbation to lysosomes function leads to in-appropriate autophagy thereby altering the autophagic flux that may leads to accumulation of autophagosomes [61, 62]. Therefore, in the present study, lysosomal membrane integrity was analysed using flow cytometry based acridine orange staining and fluorescence microscopic analysis of Lysotracker red dye. Our results showed that GCNF exposure resulted in dose dependent decrease in the acridine orange positive cells as well as decreased red fluorescence of Lysotracker red in exposed A549 cells after 24 h post treatment (Fig. 6a, b).

Next, the effect of GCNF on lysosomal pH of A549 cells was analyzed using pHrodo green dextran staining which selectively accumulates in lysosomes and its intensity is directly proportional to the pH value. Our results demonstrate a significant decrease in the intensity of pHrodo green dextran suggested loss of lysosomal acidity in GCNF treated A549 cells (Fig. 6c). We further investigated the lysosomal membrane destabilization by assaying the protein level of cathepsin D in the cytosolic fraction of GCNF treated A549 cells. A significant release of cathepsin D was observed in GCNF treated A549 cells (Fig. 6d, e). This observation strongly suggested the lysosomal destabilization following exposure to GCNF.

Since actin cytoskeleton are involved in the formation and fusion of autophagosomes with lysosomes and plays essential role in membrane rearrangement. Any damage to actin cytoskeleton can impaired the above fusion process finally leads to blockade of autophagic flux. Including this, in our study cells was found to loss their adherence property thus the effect of GCNF exposure on cytoskeleton organization was analyzed using Alexa Fluor 488 phalloidin Green staining of actin filaments. Further, immunocyto-staining of actin filaments demonstrated significant cytoskeleton disruption in GCNF exposed cells compared to control cells (Additional file 3: Figure S3).

The above observations conclusively confirmed that GCNF treatment resulted in impaired autophagy flux via lysosomal membrane destabilization and actin cytoskeleton disruption which leads to autophagosomes accumulation. Moreover, the destabilization of lysosomal membrane leads to release of cathepsin D which may further induce other form of cell death i.e. apoptosis in GCNF exposed A549 cells.

### Induction of mitochondrial damage mediated apoptosis and interlink with autophagy in GCNF exposed A549 cells

**Fig. 7:**
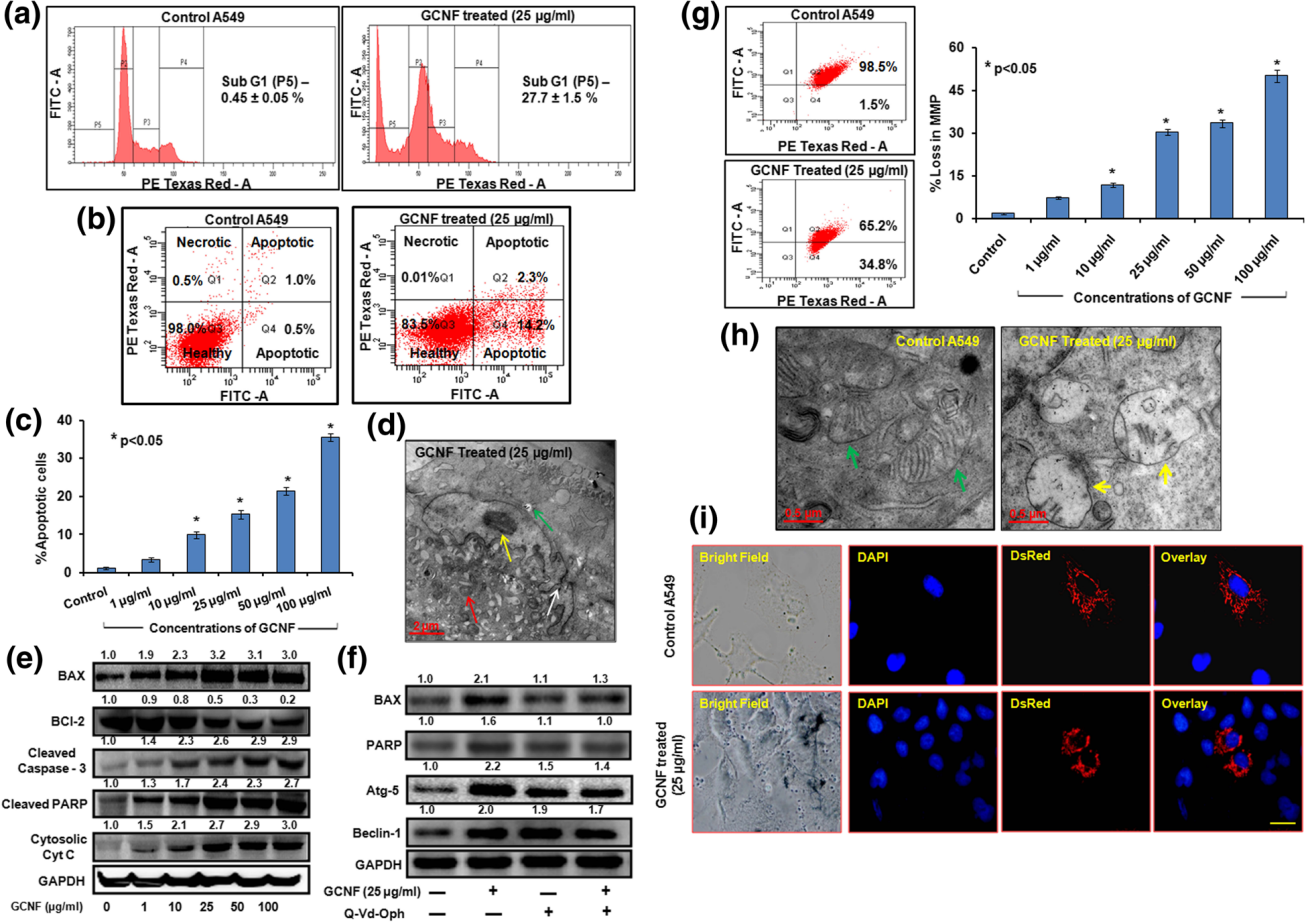
Representative flow cytograms for cell cycle analysis (**a**), annexin V/PI assay (**b**) and their statistical analysis (**c**) depicting increase in % apoptotic cells upon GCNF exposure. Values are expressed as mean ± SE of three independent experiment. *p<0.05 was considered as statistical significant. (**d**) TEM photomicrographs depicting the nuclear fragmentation (white arrow), swollen mitochondria (red arrow) and chromatin condensation (yellow arrow) which are the hall mark of apoptosis. Including this, particle accumulation (green arrow) was also found. Immunoblotting analysis of various apoptotic protein in dose dependent manner (**e**) as well as in presence of Q-Vd-OPh (caspase inhibitor) (**f**). GAPDH was used as a loading control. Densitometry analysis is represented by the digits given above the respective blots and also provided in Additional file 4: Figure S4a, S4b. (**g**) Representative flow cytograms and corresponding statistical analysis for altered mitochondrial membrane potential. Values are expressed as mean ± SE of three independent experiment. *p<0.05 was considered as statistical significant. Representative TEM photomicrographs (**h**) and corresponding fluorescence images (**i**) of A549 transfected with Mito - DsRed Plasmid showing the damage to mitochondria. In TEM, green arrow – healthy mitochondria with proper cristae whereas and yellow arrow – swollen mitochondria with dissolved cristae structure. Scale Bar – 20 μm. Per view 25 cells and 4 views per group were analyzed

Apart from its pro-survival nature autophagy has also been shown to be involved in cell death such as apoptosis by the removal of cellular constituents and survival factors resulting in the cellular toxicity [27, 63]. In the present study, the cell cycle distribution analysis demonstrated that the GCNF treatment caused a significant sub-G1 population accumulation in exposed cells (Fig. 7a) compared to control cells. This observation indicated an apoptotic mode of cell death in A549 cells that was further confirmed by annexin V/PI labeling assay. A significant increase in the population of annexin V positive cells was observed in A549 cells under similar treatment conditions (Fig. 7b, c). Moreover, as shown in Fig. 7d, the TEM analysis clearly demonstrated the fragmented nuclei, a classical hallmark of apoptotic cell death. Further the apoptotic mode of cell death was confirmed through western blot analysis of various apoptotic markers such as Bax, Bcl - 2, Caspase - 3, cytochrome c and cleaved PARP - 1. A significant increase in Bax: Bcl - 2 ratio along with increased expression level of caspase - 3 and PARP - 1 was noted in GCNF treated cells in a dose dependent manner (Fig. 7e and Additional file 4: Figure S4).

Previous studies have shown the importance of mitochondrial structure and function towards various cellular processes including apoptosis [64]. Also in our study, mitochondrial dysfunction was noticed in A549 cells which were evident from the shifting of JC -1 dye fluorescence from red (polarized mitochondria) to green (depolarized mitochondria). This conversion is the marker of collapsed mitochondrial membrane potential (MMP). Our observations on JC1 probe (a ratiometric dye) indicated a significant loss of MMP (30.3%, 33.5%, and 50.1% at 25 μg/ml, 50 μg/ml, and 100 μg/ml respectively) in GCNF treated A549 cells when compared with control cells (Fig. 7g). This result was further corroborated by the analysis of A549 cells which were transient transfected with Mito - DsRed plasmid. There was an intact filament structure of mitochondria found in untreated cells whereas cells exposed to GCNF showed a disrupted structure as shown in Fig. 7i. These results were also confirmed by the western blot analysis of cytochrome-c (an indicator of intrinsic apoptosis through mitochondrial damage) showed an increased expression level in the cytosol of GCNF exposed cells (Fig. 7e). Thus our results conclude the role of mitochondrial damage in apoptotic cell death upon GCNF exposure to A549 cells.

Next, to investigate the effect of autophagy on viability as well as apoptosis induction after GCNF exposure, A549 cells were pre-incubated with 3 - Methyladenine (3MA; an autophagy inhibitor) and siRNA for LC3 and subjected to MTT assay and western blot analysis of apoptosis as well as autophagic proteins. Interestingly, autophagy suppression resulted in significant down regulation of apoptotic proteins (Bax and PARP – 1) as well as attenuate the cell death in GCNF treated A549 cells (Additional file 5: Figure S5a, S5b). The suppression of autophagy was confirmed by the decreased expression level of LC3 and Beclin-1 in 3MA and siRNA treated cells. This observation suggested that GCNF mediated autophagy was an upstream event that finally culminated into apoptotic cell death.

### Loss of ATP level and glucose uptake deprivation in GCNF exposed A549 cells

Since damage to mitochondria may cause energy deprivation which may leads to the induction of apoptosis and other forms of cell death. In the present study results also demonstrated that there was a significant reduction in ATP level of GCNF exposed cells compared to control cells (Additional file 6: Figure S6a). Further, the glucose uptake by GCNF exposed A549 cells were also found to repress in a concentration dependent manner (Additional file 6: Figure S6b). Thus the results suggested the energetic impairment in GCNF exposed cells.

### Induction of oxidative stress and their role in autophagy, apoptosis as well as genotoxicity induction

**Fig. 8:**
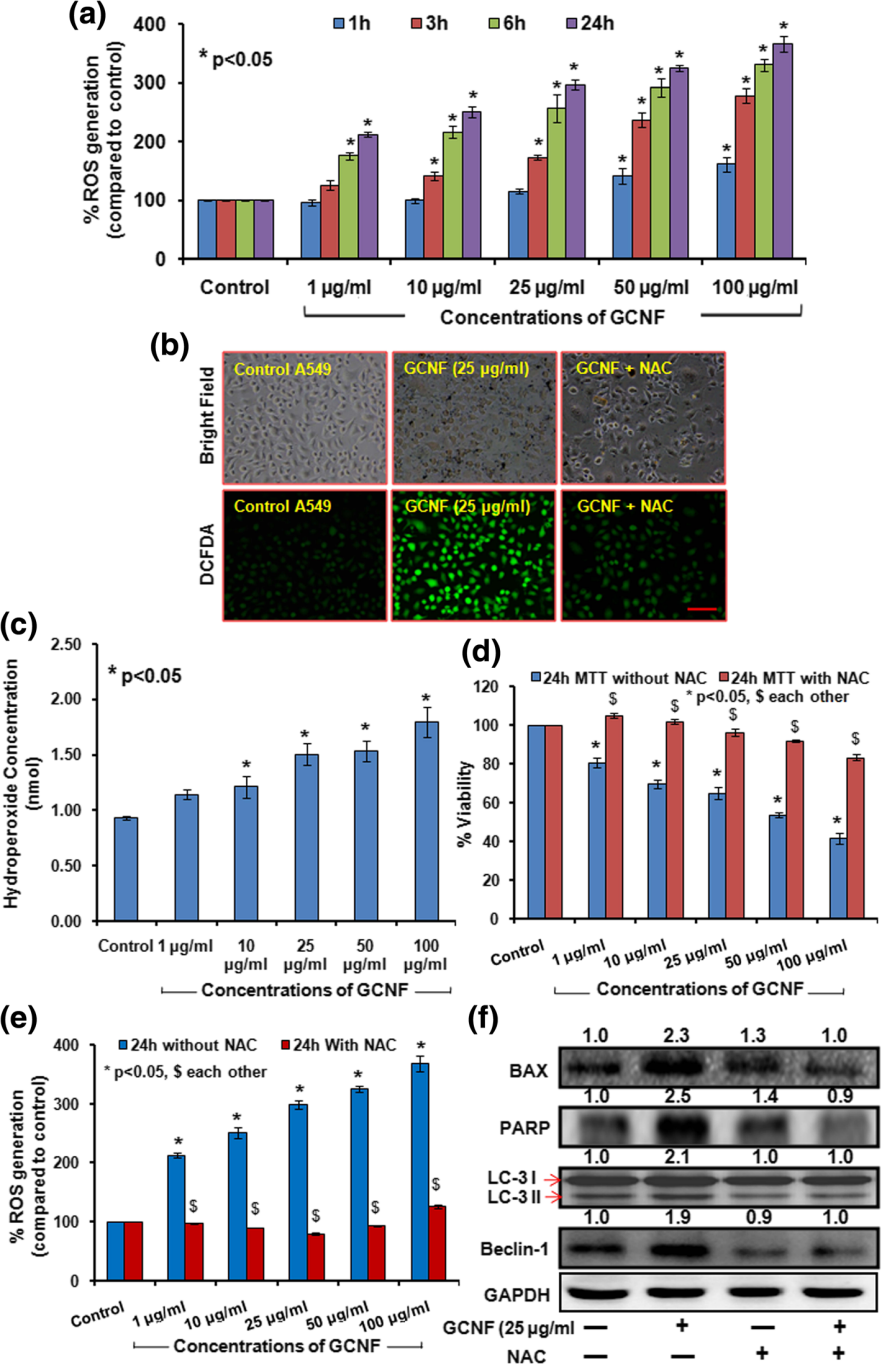
GCNF induced significant dose and time dependent increase in ROS production depicted by the increase in DCFDA fluorescence measured through plate reader (**a**) and respective fluorescence images (**b**). Scale Bar – 300 μm. Per view 100 cells and 4 views per group were analyzed. (**c **) Effect of GCNF on lipid peroxidation (LPO) was monitored compared to control cell. (**d**, **e**) NAC (ROS scavenger) have significant impact on viability of GCNF exposed A549 cells as well as ROS production in GCNF exposed A549 cells. Values are expressed as mean ± SE of three independent experiment. *p<0.05 was considered as statistical significant. (**f**) Immunoblotting analysis of apoptotic as well as autophagic protein in presence and absence of NAC after GCNF exposure in A549 cell. GAPDH was used as an internal control. Densitometry analysis is represented by the digits given above the respective blots and also provided in Additional file 7: Figure S7. Values are expressed as mean ± SE of three independent experiment. *p<0.05 was considered as statistical significant

Oxidative stress has shown to play a major role in NM induced toxicity [65, 66]. In the present study, GCNF exposure resulted into a significant increase in reactive oxygen species (ROS) which was evident through increased fluorescence of DCFDA in exposed A549 cells compared to control cells (Fig. 8a, b). A significant increase in the DCF intensity was noted in these cells in a dose as well as time dependent manner. An increase of 257%, 292%, 331% at 25, 50 and 100 μg/ml was noted at 6 h post treatment which was further enhanced to 298%, 325% and 368% at 24 h post treatment (Fig. 8a). To study the effect of GCNF exposure on redox status of A549 cells, lipid peroxidation (LPO) level was evaluated using TBARS assay which showed a dose dependent increase in malondialdehyde (MDA) content, marker of LPO under similar treatment conditions (Fig. 8c).

In previous studies, ROS has been shown to play a pivotal role in apoptosis as well as autophagy induction in NM exposed cells [65, 67]. Next, we investigated the contribution of GCNF induced ROS in autophagosomes accumulation, apoptosis induction and ultimately in cell death by using N-acetyl L-cysteine (NAC; a ROS scavenger). Interestingly, in our study, supplementation with NAC resulted in suppression of GCNF mediated autophagy as evident by protein level of LC3 - II and Beclin - 1. The expression of these markers was comparable to untreated control (Fig. 8f and Additional file 7: Figure S7). Moreover, NAC supplementation also repressed the protein expression of Bax and PARP - 1 (Fig. 8f) as compared to GCNF treated cells. In the presence of NAC, cell viability also increased which was comparable to control cells in GCNF exposed cells. Similarly, the effect of NAC on ROS production was quite evident through the decreased DCFDA fluorescence in NAC pre-treated cells. These observations suggested that ROS could play a critical role in GCNF inducing cancer cell death via autophagic-apoptotic axis.

It has been shown that any imbalance between ROS level and antioxidant level may lead to genomic instability [68], so we assessed the effect of GCNF induced ROS on the integrity of A549 DNA using Comet assay and micronucleus assay. It was demonstrated that there was a significant increase in Olive tail moment as well as number of MN with the increase in concentrations of GCNF (Additional file 8: Figure S8a, S8b). The amount of DNA damage was higher in Fpg modified Comet assay compared to alkaline Comet assay proving the oxidative stress induced DNA damage. Thus our results showed that graphite carbon nanofibers may also exert genomic in-stability after their interaction with biological systems.

## Discussion

Although CBNM and especially fibrous carbon nanomaterials (CNT and GCNF) have unlimited potential for myriad of applications but their successful implementation will require strict safety assurance to human and environment. Among them, GCNF have gained significant attention and replaced the CNT for various applications due to their superior physico-chemical properties. Apart from increased uses of GCNF, toxicity evaluation is of great necessity in order to enable them for biological applications or for developing nanobased products for consumer needs. Data in literature regarding the toxicity potential of GCNF is limited which inhibit the regulatory authorities to make consensus regarding their safer use in biomedical applications. Till now only three in vivo studies have been conducted which demonstrate the pulmonary toxicity potential of GCNF, mediated through inflammation, fibrosis, granulomas formation [69–71]. Including this, there is a contrasting data is available regarding in vitro toxicity potential of GCNF with the underlying molecular mechanism is largely unknown [23, 24].

The dimension and high aspect ratio of CNT and GCNF makes them similar to asbestos fibers which is a known carcinogen [17]. Due to this similarity, fibrous carbon nanomaterials come into the scrutiny of regulatory authorities for their toxicity evaluation. In occupational settings, inhalation is the primary route for fibrous nanomaterials to get deposited into the alveolar cells which may cause damage to lung function and leads to systemic toxicity [15]. The World Health Organization has also defined the characteristics of respirable fiber with length greater than 5 μm and a diameter of less than 3 μm [16]. The toxicity of fibrous nanomaterials largely depends upon their length, biopersistance, chemical composition and respirability. Upon interaction with cells, CNT has been shown to produce oxidative stress, inflammation, fibrosis, carcinogenesis etc. [18–22]. But the same relevant information regarding GCNF interaction with biological systems is largely unknown. In the present study, TEM analysis of GCNF confirms their fibrous nature with respirable size limit implicating the possible threat of inhalation toxicity. Also, our DLS analysis showed a stable dispersion of GCNF in culture medium as depicted by zeta potential. Thus based on characterization it was implied that GCNF can cause similar adverse effects to CNT and asbestos which should be properly characterized given their widespread use. Further, TEM EDAX analysis was employed to analyze the presence of metal impurities in sample. Our data showed that GCNF contain very negligible amount of aluminum as a metal content which confirmed that any adverse effect associated with GCNF exposure is solely because of fibers not by impurities.

For designing suitable nano-based drug delivery system, the cellular internalization of NM is a critical issue. Our results of flow cytometry and TEM analysis showed significant internalization of GCNF in the cytoplasm as well as in enclosed form in vesicle of A549 cells. Our results were in accordance to the previous results showing the dose-dependent internalization of carbon nanofibers in cells [23]. It has been showed that after cellular internalization CNT can pose adverse effects to DNA, protein, and mitochondria and simultaneously can induce cell death either through autophagy, apoptosis or necrosis [2, 19, 72, 73]. Including this various other types of NM have been shown to induce autophagy in mammalian cell lines because of their identification as foreign particle, pathogen or protein by the cells [26, 30, 74]. Autophagy acts as a cardinal process to sequester and degrade these materials to maintain cellular homeostasis. Recently the role of autophagic pathway of cell death has also been shown in NM induced toxicity [30]. During autophagy, the formation of autophagosomes (double membrane autophagic vacuole) and the conversion of LC3 – I to LC3 - II are the hallmarks of autophagic process [53]. Since in the present study, GCNF was found intracellular in enclosed form in a vesicle and it has been reported that the vesicle with foreign materials merge with lysosomes to degrade their enclosed materials and this process is closely linked with autophagy also [30]. This prompt us to investigate the role of autophagy in GCNF induced cell death and interestingly the above autophagy related phenomena were clearly evident in the GCNF exposed A549 cells which was in accordance to the previous results for CBNM [31].

The accumulation of autophagosomes can result either from enhanced induction of autophagy or by blockade of autophagic flux [53]. During induction of autophagy, LC3 – II is degraded by the autolysosomes which inhibited during blockade of autophagic flux [53]. Thus by comparing the amount of LC3 - II in the presence and absence of lysosomes degradation one can differentiate between these two possible phenomena. In our study we also found the disruption of autophagic flux evident through the accumulation of SQSTM/p62 – essential for cargo recognition and specifically degrade at later stage of autophagy. Various signaling pathways have been shown to regulate autophagy, out of them PI3K/Akt/mTOR pathway is a classical pathway in which mTOR (mammalian target of rapamycin) gets inhibited leading to enhanced autophagy induction [54]. However, apart from classical role of mTOR, it has also been reported that autophagy induction could be an mTOR independent process and inhibition of mTOR could disturbed the autophagic flux disruption further may leads to enhanced autophagosomes accumulation rather than autophagy induction [55–60]. Also in the present study, the accumulation of autophagosomes was found to be mediated through the classical mTOR pathway which was in accordance to the previously reported results.

For the successful degradation of autophagic substrate, autophagic flux needs to be regulated in a positive direction i.e. fusion of autophagosomes with lysosomes to their complete degradation. Any perturbation to lysosomes function leads to inappropriate autophagy thereby altering the autophagic flux that may leads to accumulation of autophagosomes [61, 62]. Previous studies have shown the relevance of lysosomal dysfunction in different NM exposed cells which have been implicated in disease pathogenesis [30]. There are two type’s lysosomal membrane destabilization named lysosomal membrane permeabilization (LMP) and lysosomal membrane rupture (LMR) have suggested in NM induced toxicity [75, 76]. Both differ in the extent of lysosomal membrane damage and the later has been shown to be implicated in various neurodegenerative disorders [77]. In addition, LMP and LMR have been shown to induce other forms of cell death including apoptosis as a consequence of membrane damage leading to release of lysosomal enzymes [75]. Additionally, our results also demonstrated the significant lysosomal impairment manifested as compromised lysosomal membrane, decreased lysosomal pH and release of lysosomal cathepsin D. Including this, we also found the disruption of cytoskeleton in GCNF exposed cells compared to control cells. The actin cytoskeleton is involved in the formation and fusion of autophagosomes with lysosomes and plays essential role in membrane rearrangement [78]. The disruption of cytoskeleton can be a result of lysosomal membrane rupture (LMR) and previous reports have also shown the NM induced cytoskeleton disruption in cultured cells [76, 78]. Thus our results were in agreement to the previous results and showing the potential of GCNF to induce autophagosomes accumulation due to impaired autophagic flux via disruption of lysosomes and cytoskeleton which is similar to other CBNM induced toxicity [31, 76].

Since autophagic flux blockade can results into the activation of autophagic cell death as previously reported due to the insufficient energy supply accompanied by decrease recycling of damaged proteins [79]. Also in our study, we found that the autophagosomes accumulation was mainly induced at those concentrations which were toxic to the cells suggesting the role of autophagy in GCNF induced cell death. This was further confirmed by the pretreatment of A549 cells with 3 - MA (autophagy inhibitor) prior to GCNF exposure which leads to attenuation of cell death in GCNF exposed A549 cells. Release of lysosomal enzyme, cathepsin D, into the cytoplasm have shown to induce apoptotic cell death [80]. Also, the autophagy contributes to cell death through apoptosis by the removal of cellular constituents and survival factors resulting in the cellular toxicity [63, 81]. In our study, we also found a significant induction of typical apoptotic cell death confirmed by the TEM analysis and flow cytometric observations of Annexin V/PI positive cells after GCNF exposure which was mediated through the mitochondrial damage and cell cycle arrest. Our results were in concurrent with the previous studies which have shown the importance of mitochondrial structure and function towards various cellular processes including apoptosis [64].

Previous studies have shown the interplay between apoptosis and autophagy suggesting they might precede each other, coexist or mutually exclusive [82–84]. Further, in our study by blocking the autophagosomes formation there was a reduction in the number of apoptotic cells and subsequent cell death. Therefore, it was suggested that GCNF mediated autophagy initiated earlier than the apoptosis and the autophagic perturbation resulted in apoptotic cell death as reported previously for CNT exposed cells.

Oxidative stress has been shown to play a important role in NM induced toxicity [65]. Also the critical role of intracellular ROS has been highlighted in the CBNM including CNT toxicity [79]. In the present study, GCNF was found to induce concentration and time dependent ROS production with damage to cellular redox state as similarly to the previously reported results. The association of ROS with apoptosis or autophagy or even both cellular death processes has been demonstrated [65, 67]. We also found that the induction of both cell death processes i.e. apoptosis and autophagy in the present study were dependent upon the ROS involvement. In the presence of NAC, there was a significant decrease in the presence of LC-3 positive cells along with decreased expression level of apoptotic as well as autophagic proteins with subsequent cell death. These observations suggested that ROS could play a critical role in GCNF inducing cell death via autophagic-apoptotic axis.

## Conclusions

**Fig. 9:**
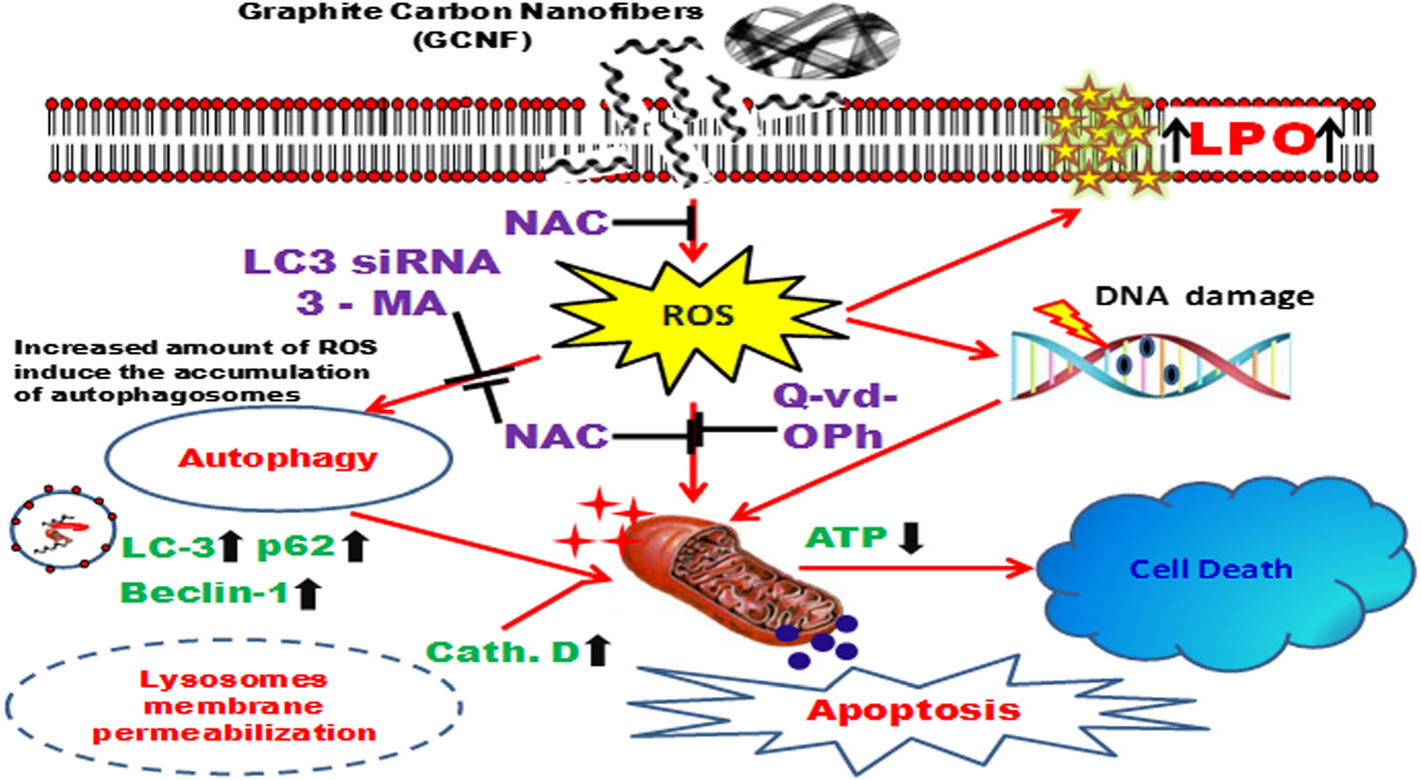
Graphical diagram representing the mechanism of cell death in A549 cells by graphite carbon nanofibers. Exposure to GCNF causes the increased production of reactive oxygen species which activates the autophagosomes accumulation, apoptosis induction as well as damage to DNA. Further mechanistic studies revealed the interconnection between apoptosis and autophagy through the impairment of lysosomes and energy pool. Thus GCNF induced nanotoxicity through modulation of autophagy apoptosis axis via oxidative stress

In the present study, we reported the interaction of graphite carbon nanofibers (GCNF) with human lung adenocarcinoma (A549) cells accompanied by interlink between autophagy, apoptosis and oxidative stress. All considered, our data suggest that GCNF triggered the oxidative stress dependent lysosomes and cytoskeleton disruption which leads to autophagic dysregulation in A549 cells. This dysregulation governs the pathogenesis of GCNF induced cell death in the form of apoptosis mediated through energetic impairment and damage to DNA, mitochondria. The significant results reported in our study suggest that the generations of reactive oxygen species are the earliest events that results in dysregulation of autophagy followed by mitochondrial damage mediated apoptosis and ultimately cell death as summarized in Fig. 9. Our results have significant impact due to the occupational exposure of GCNF warranted their evaluation prior to use and also signify the importance of these materials in the biomedical applications utilizing their autophagy disruption capacity. This provides new insights for potential medical applications of GCNF but the need of the hour is to evaluate the fate of these materials in in vivo system for their safe biomedical applications.

## Additional files

Additional file 1: Figure S1. Uptake mechanism of GCNF in A549 cells. (a) Effect of specific endocytosis and phagocytosis inhibitor on GCNF internalization in cultured A549 cells. Cells were incubated with each inhibitor for 2 h and further exposed to GCNF (25 μg/ml) for additional 24 h. A significant decrease in uptake of GCNF was found in cells incubated at 4 ^0^C and with sodium azide and amiloride showing the uptake mediated through energy dependent endocytosis. Wortmanin treatment does not affect the internalization which depicts the absence of phagocytosis pathway. (b) Endocytosis was found to be mediated through clathrin as the treatment of sucrose and chlorpromazine reduce the uptake of GCNF in A549 cells. Values are expressed as mean ± SE of three independent experiment. *p<0.05 was considered as statistical significant. (PDF 68 kb)

Additional file 2: Figure S2. Densitometry analysis of autophagic proteins in dose dependent (a) and time dependent (b) manner analyzed through western blot. GAPDH was served as a loading control. Values are expressed as mean ± SE of three independent experiment. *p<0.05 was considered as statistical significant. (c) Real Time PCR for specificity of LC3 siRNA at gene level. (d) mRNA expression profile of control and GCNF exposed A549 cells for various genes involved in autophagy and apoptosis induction. The data represent the mean ± SE of three independent experiment. *p<0.05was considered as statistical significant. (PDF 930 kb)

Additional file 3: Figure S3. Cytoskeleton disruption in GCNF exposed A549 cells. Representative fluorescence photomicrographs of GCNF exposed A549 cells stained with Oregon green 488 phalloidin for actin fibers showed a significant damage compared to control cells. Per view 6 cells and 4 views per group were analyzed. Scale Bar – 20 μm. (PDF 662 kb)

Additional file 4: Figure S4. Densitometry analysis of various apoptotic proteins in dose dependent manner (a) as well as in presence of Q-Vd-OPh (caspase inhibitor) (b). GAPDH was used as a loading control. Values are expressed as mean ± SE of three independent experiment. *p<0.05 was considered as statistical significant. (PDF 660 kb)

Additional file 5: Figure S5. GCNF induced autophagy mediated apoptosis in A549 cells. (a) Immunoblotting and respective densitometry analysis of apoptotic as well as autophagic protein in the presence of 3 – methyladenine (3 - MA) after GCNF (25 μg/ml) exposure in A549 cells. GAPDH was used as loading control. (b) Immunoblotting analysis for specificity of LC siRNA at protein level. (c) Immunoblotting and respective densitometry analysis of apoptotic as well as autophagic protein in the presence of LC3 siRNA after GCNF (25 μg/ml) exposure in A549 cells. GAPDH was used as loading control. Values are expressed as mean ± SE of three independent experiment. *p<0.05 was considered as statistical significant. (d) Viability of GCNF exposed cells was assessed using MTT assay in the presence or absence of 3-MA to check the role of autophagy in cell death. Values are expressed as mean ± SE of three independent experiment. *p<0.05 was considered as statistical significant. (PDF 1330 kb)

Additional file 6: Figure S6. GCNF induced ATP loss and inhibition of glucose uptake in A549 cells. (a) A549 cells exposed to GCNF (1 – 100 μg/ml) for 24 h time period showed a dose dependent decrease in ATP level compared to control cells which was measured using ATP measurement kit by a luminometer. Values are expressed as mean ± SE of three independent experiment. *p<0.05 was considered as statistical significant. (b) A549 cells were treated with GCNF for 24 h time and glucose uptake was determined using 2 - NBDG according to the manufacturer’s instruction. Significant reduction in fluorescence was detected as measured by the flow cytometer. (PDF 913 kb)

Additional file 7: Figure S7. Densitometry analysis of apoptotic as well as autophagic protein in presence and absence of NAC after GCNF (25 μg/ml) exposure in A549 cell. GAPDH was used as an internal control. Values are expressed as mean ± SE of three independent experiment. *p<0.05 was considered as statistical significant. (PDF 290 kb)

Additional file 8: Figure S8. GCNF induced DNA damage and chromosomal breakage in A549 cells. (a) Assessment of DNA damage by GCNF in A549 cells was carried out using Comet assay (Standard as well as Fpg modified) after 6 h of exposure. Results showed a significant increase in OTM value (Comet parameter) of exposed cells compare to control cells. Values are expressed as mean ± SE of three independent experiment. *p<0.05 was considered as statistical significant. # when compared to standard alkaline Comet assay. (b) Flow cytometry based micronucleus (MN) assay showed a significant increase in MN formation with increasing concentration of GCNF after 3 h and 6 h exposure. This depicts the induction of chromosomal breakage in A549 cells after GCNF exposure. Values are expressed as mean ± SE of three independent experiment. *p<0.05 was considered as statistical significant. (PDF 674 kb)

## Abbreviations

CBNM: Carbon based nanomaterials
DCFDA: 2′,7′-Dichlorofluorescein diacetate, Fpg, formamidopyrimidine DNA glycosylase
DLS: Dynamic light scattering
DMEM – F 12: Dulbecco’s modified eagle medium: nutrient mixture F-12 (Ham) (1 : 1) powder
DMSO: Dimethyl sulfoxide
EtBr: Ethidium bromide
GCNF: Graphite carbon nanofibers
JC - 1: 5,5’, 6,6’-Tetrachloro-1,1’,3,3’-tetraethyl- benzimidazolecarbocyanine iodide dye
MDC: Monodansyl cadaverine
MTT: 3-(4,5-dimethylthiazol-2-yl)-2,5-diphenyltetrazolium bromide
NaCl: Sodium chloride
NM: Nanomaterials
PBS: Phosphate buffered saline
ROS: Reactive oxygen species
SEM: Scanning electron microscopy
TEM: Transmission electron microscopy
