# Supplementary figures and images for: Impaired lysosomal activity mediated autophagic flux disruption by graphite carbon nanofibers induce apoptosis in human lung epithelial cells through oxidative stress and energetic impairment
Source: Part Fibre Toxicol. 2017 Apr 28;14:15. doi: 10.1186/s12989-017-0194-4 (PMC5408471; doi:10.1186/s12989-017-0194-4)

Figure S1

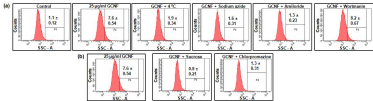

Supplement: Supplementary file 1 — Uptake mechanism of GCNF in A549 cells. (a) Effect of specific endocytosis and phagocytosis inhibitor on GCNF internalization in cultured A549 cells. Cells were incubated with each inhibitor for 2 h and further exposed to GCNF (25 μg/ml) for additional 24 h. A significant decrease in uptake of GCNF was found in cells incubated at 4 0C and with sodium azide and amiloride showing the uptake mediated through energy dependent endocytosis. Wortmanin treatment does not affect the internalization which depicts the absence of phagocytosis pathway. (b) Endocytosis was found to be mediated through clathrin as the treatment of sucrose and chlorpromazine reduce the uptake of GCNF in A549 cells. Values are expressed as mean ± SE of three independent experiment. *p<0.05 was considered as statistical significant. (PDF 68 kb) [file 12989_2017_194_MOESM1_ESM.pdf]

**Figure S2**

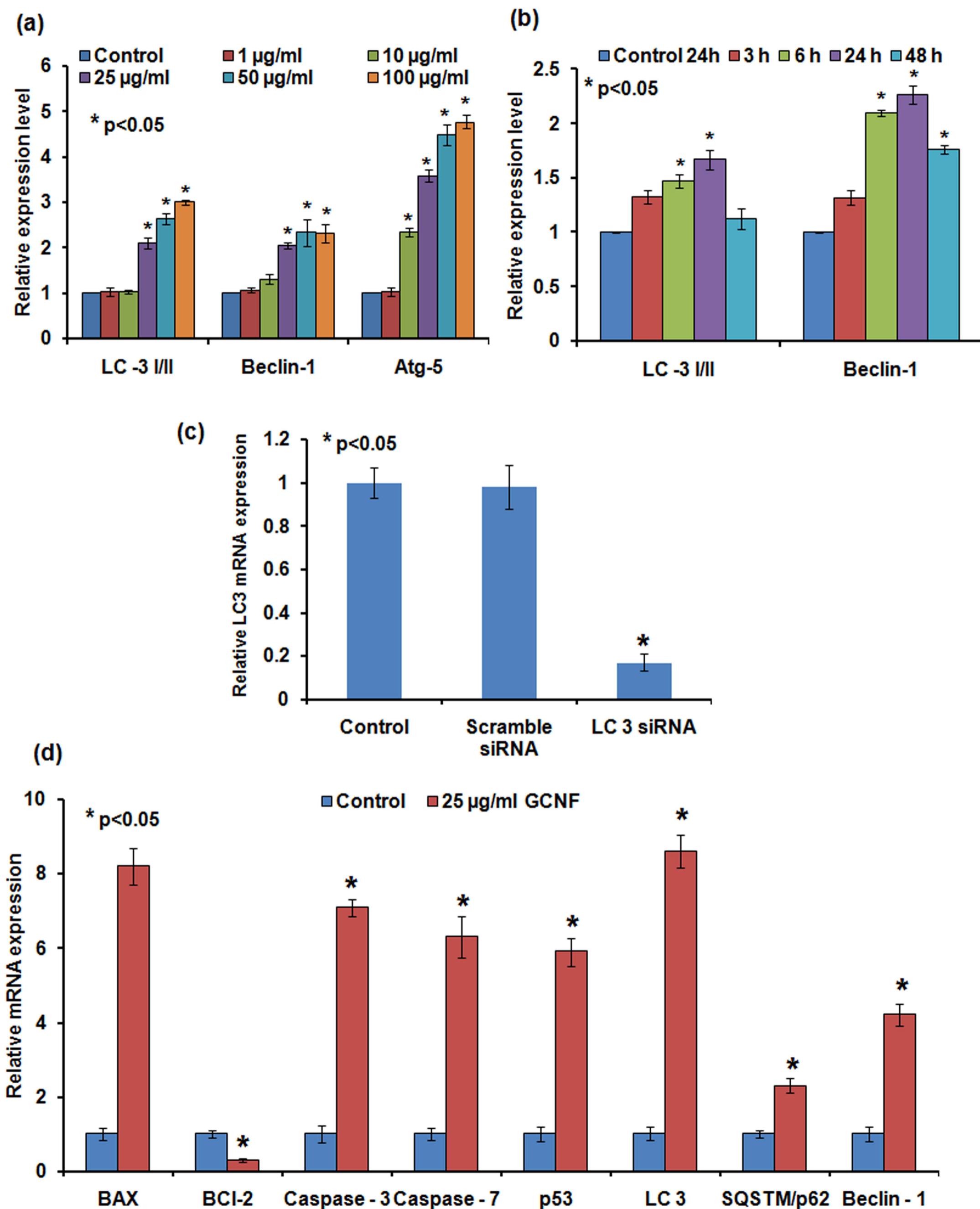

Supplement: Supplementary file 2 — Densitometry analysis of autophagic proteins in dose dependent (a) and time dependent (b) manner analyzed through western blot. GAPDH was served as a loading control. Values are expressed as mean ± SE of three independent experiment. *p<0.05 was considered as statistical significant. (c) Real Time PCR for specificity of LC3 siRNA at gene level. (d) mRNA expression profile of control and GCNF exposed A549 cells for various genes involved in autophagy and apoptosis induction. The data represent the mean ± SE of three independent experiment. *p<0.05was considered as statistical significant. (PDF 930 kb) [file 12989_2017_194_MOESM2_ESM.pdf]

**Figure S3**

Control  
A549

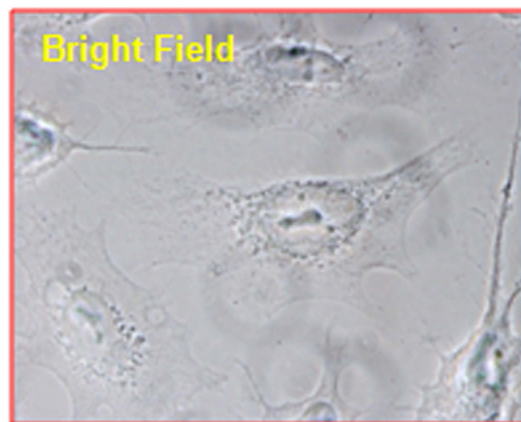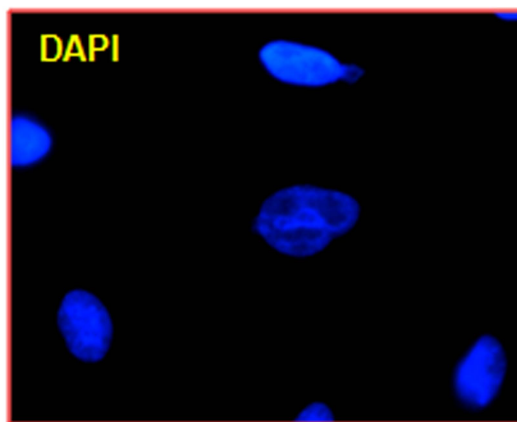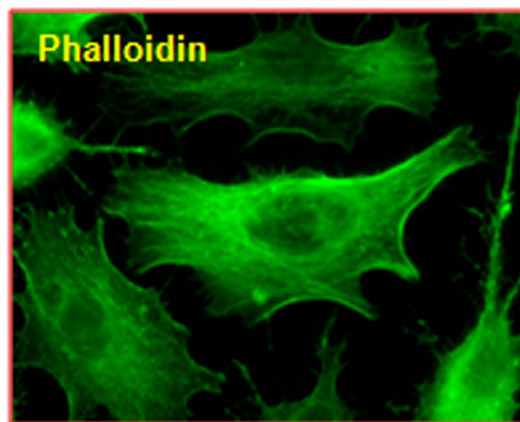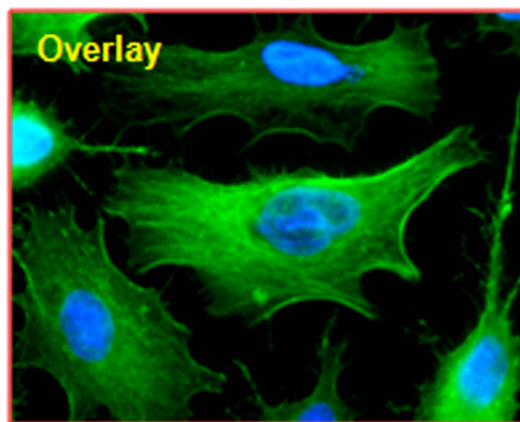

GCNF treated  
(25  $\mu$ g/ml)

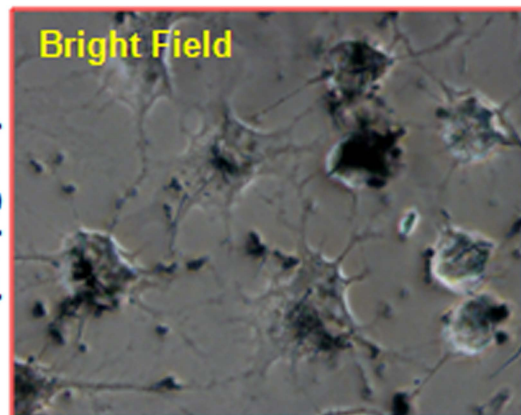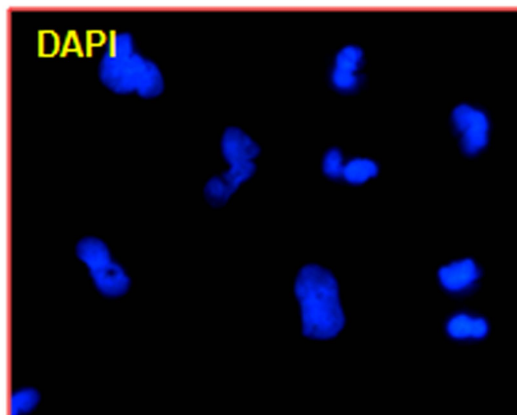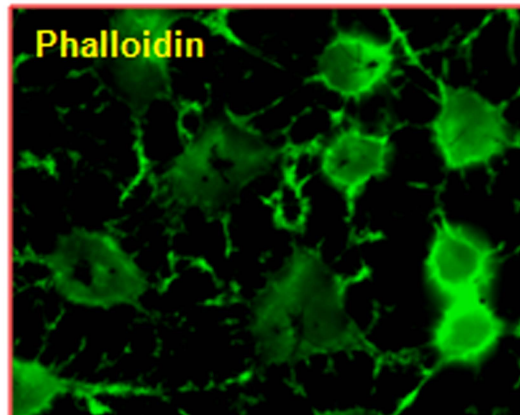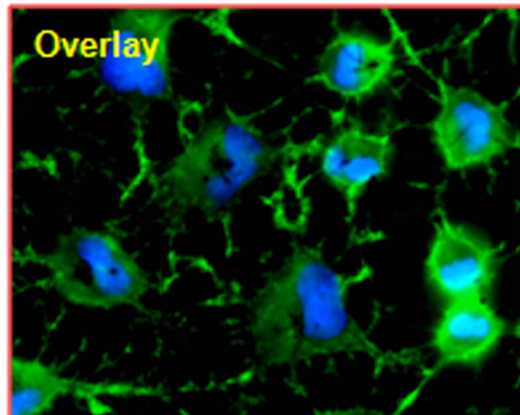

Supplement: Supplementary file 3 — Cytoskeleton disruption in GCNF exposed A549 cells. Representative fluorescence photomicrographs of GCNF exposed A549 cells stained with Oregon green 488 phalloidin for actin fibers showed a significant damage compared to control cells. Per view 6 cells and 4 views per group were analyzed. Scale Bar – 20 μm. (PDF 662 kb) [file 12989_2017_194_MOESM3_ESM.pdf]

**Figure S4**

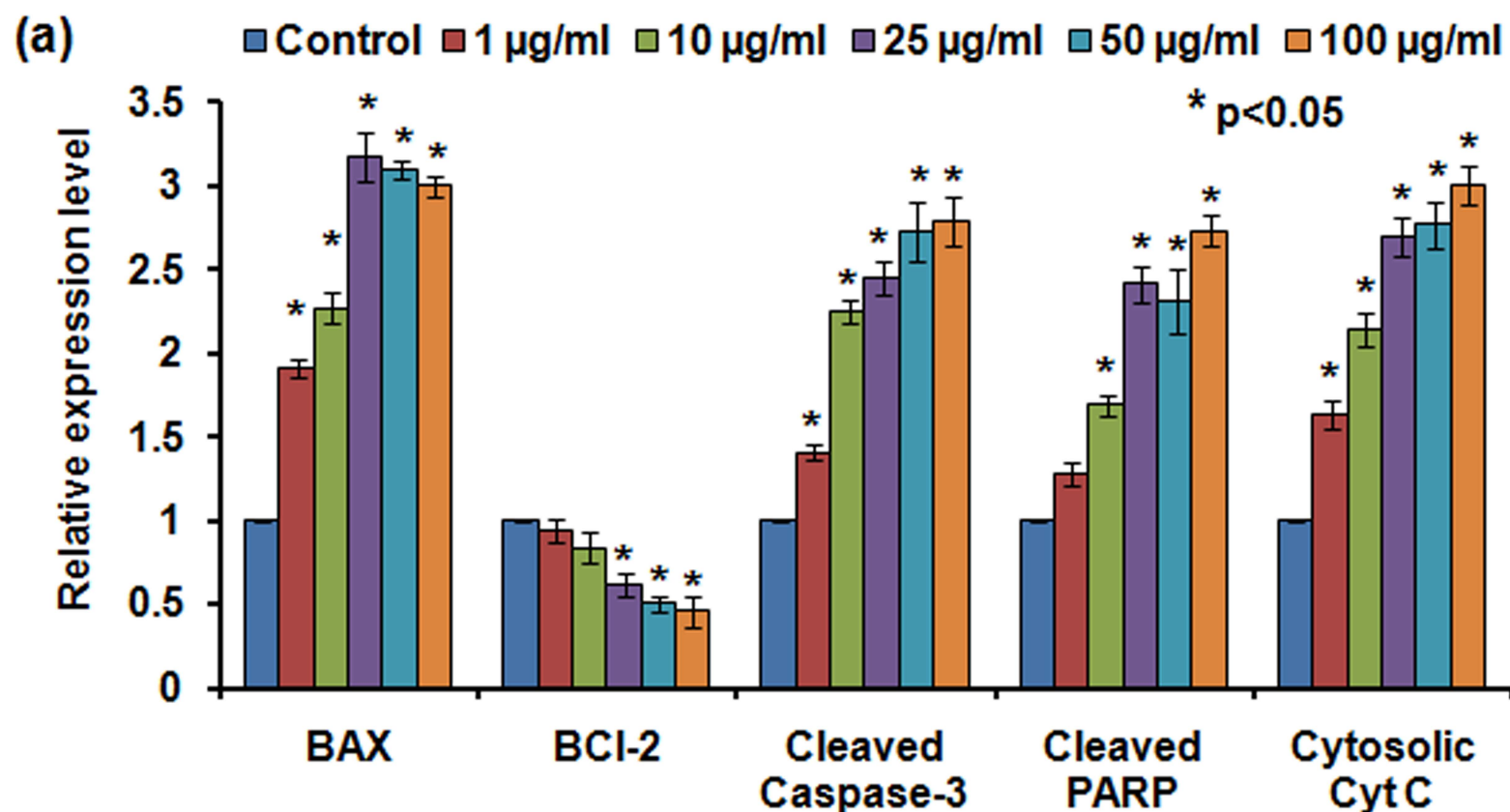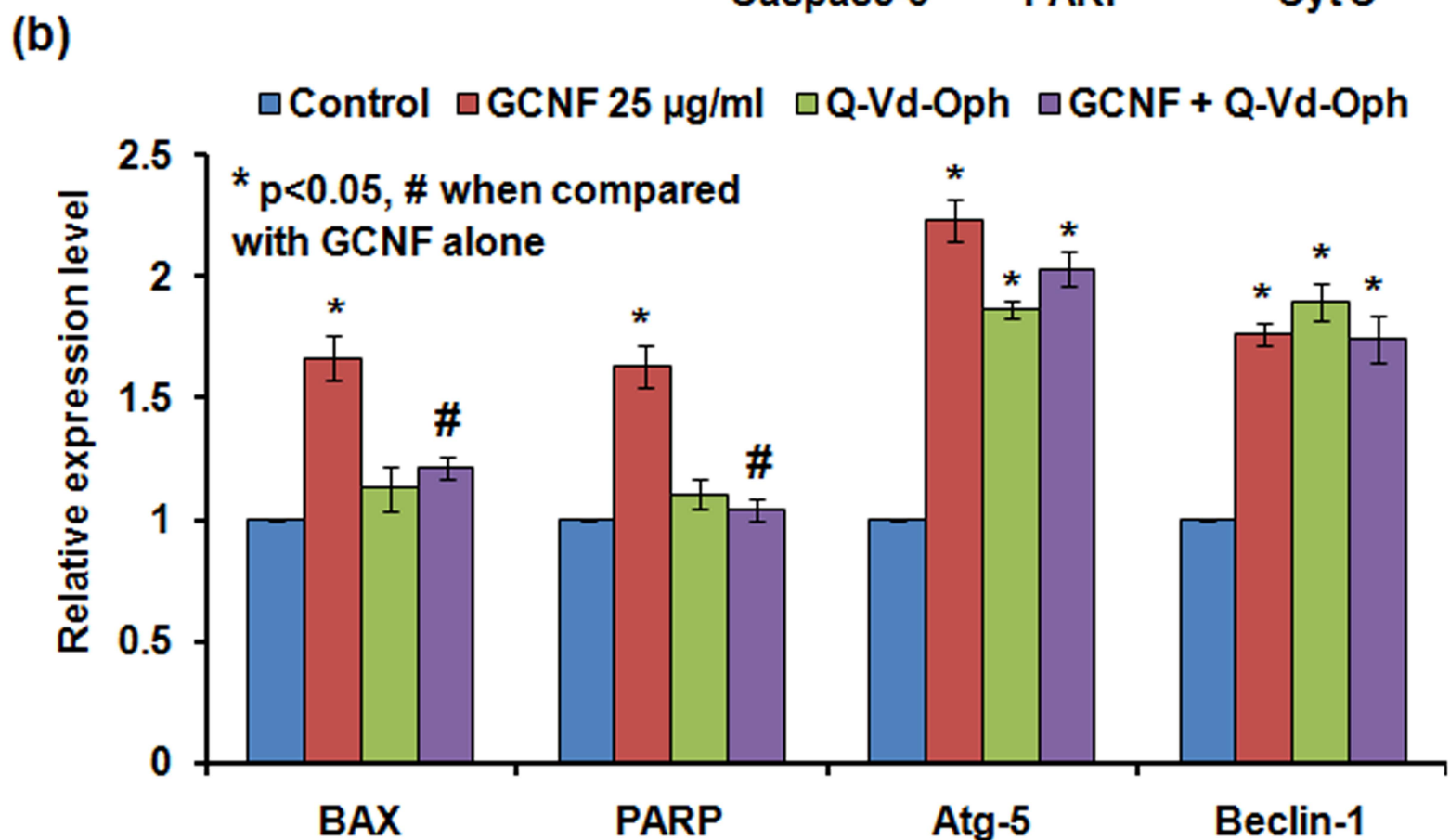

Supplement: Supplementary file 4 — Densitometry analysis of various apoptotic proteins in dose dependent manner (a) as well as in presence of Q-Vd-OPh (caspase inhibitor) (b). GAPDH was used as a loading control. Values are expressed as mean ± SE of three independent experiment. *p<0.05 was considered as statistical significant. (PDF 660 kb) [file 12989_2017_194_MOESM4_ESM.pdf]

**Figure S5**

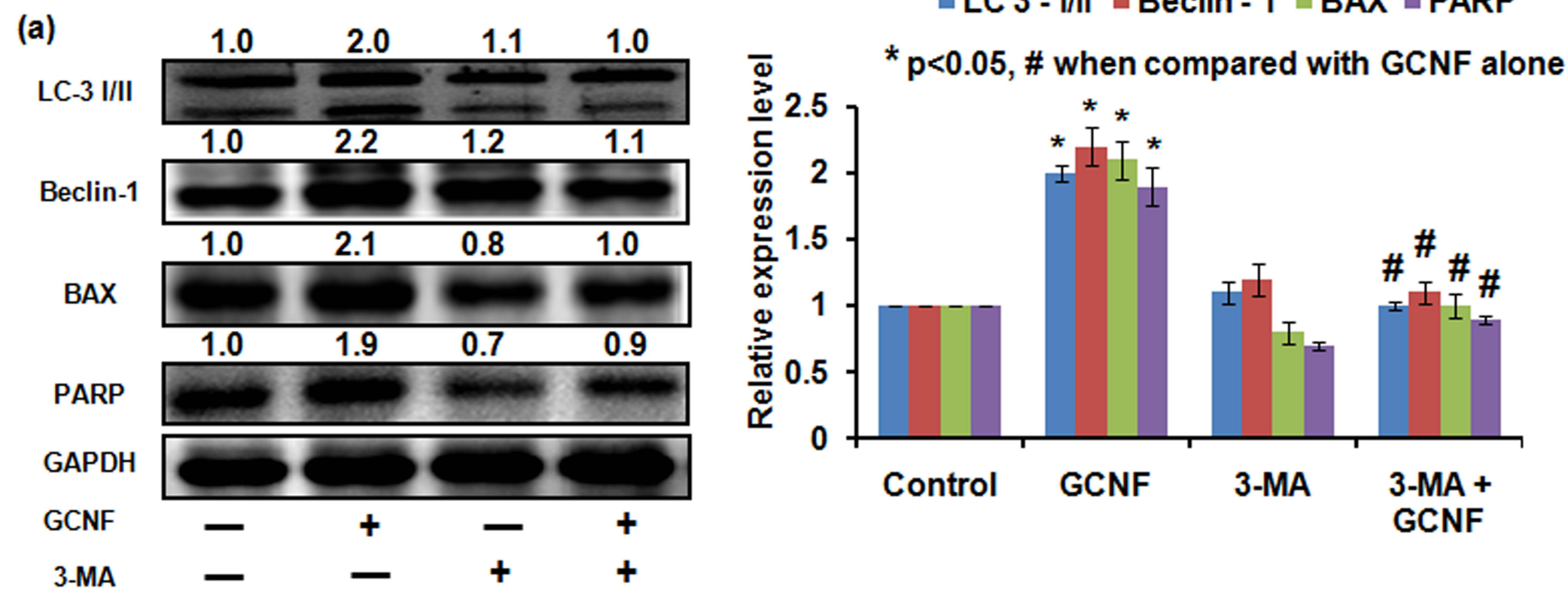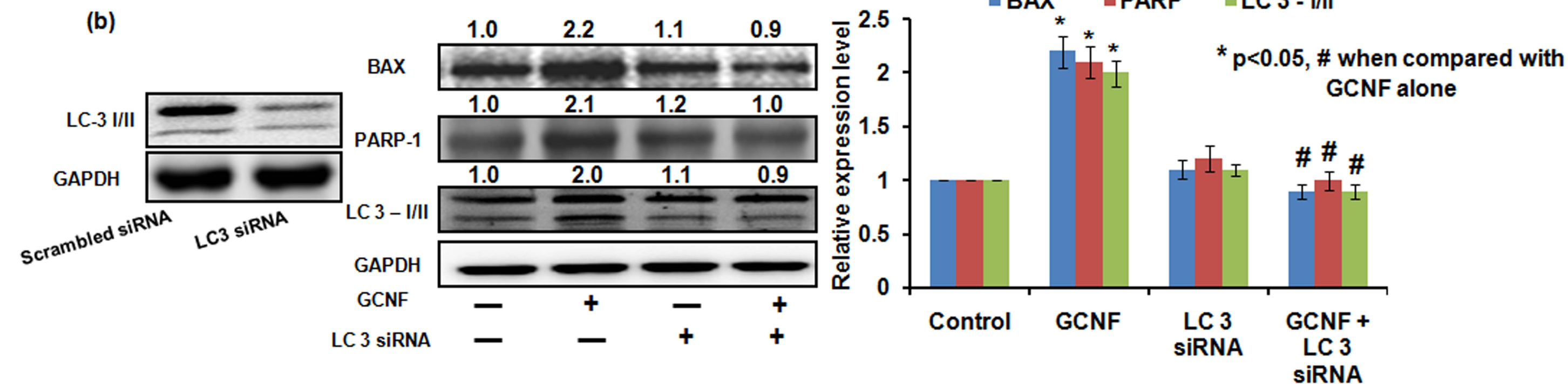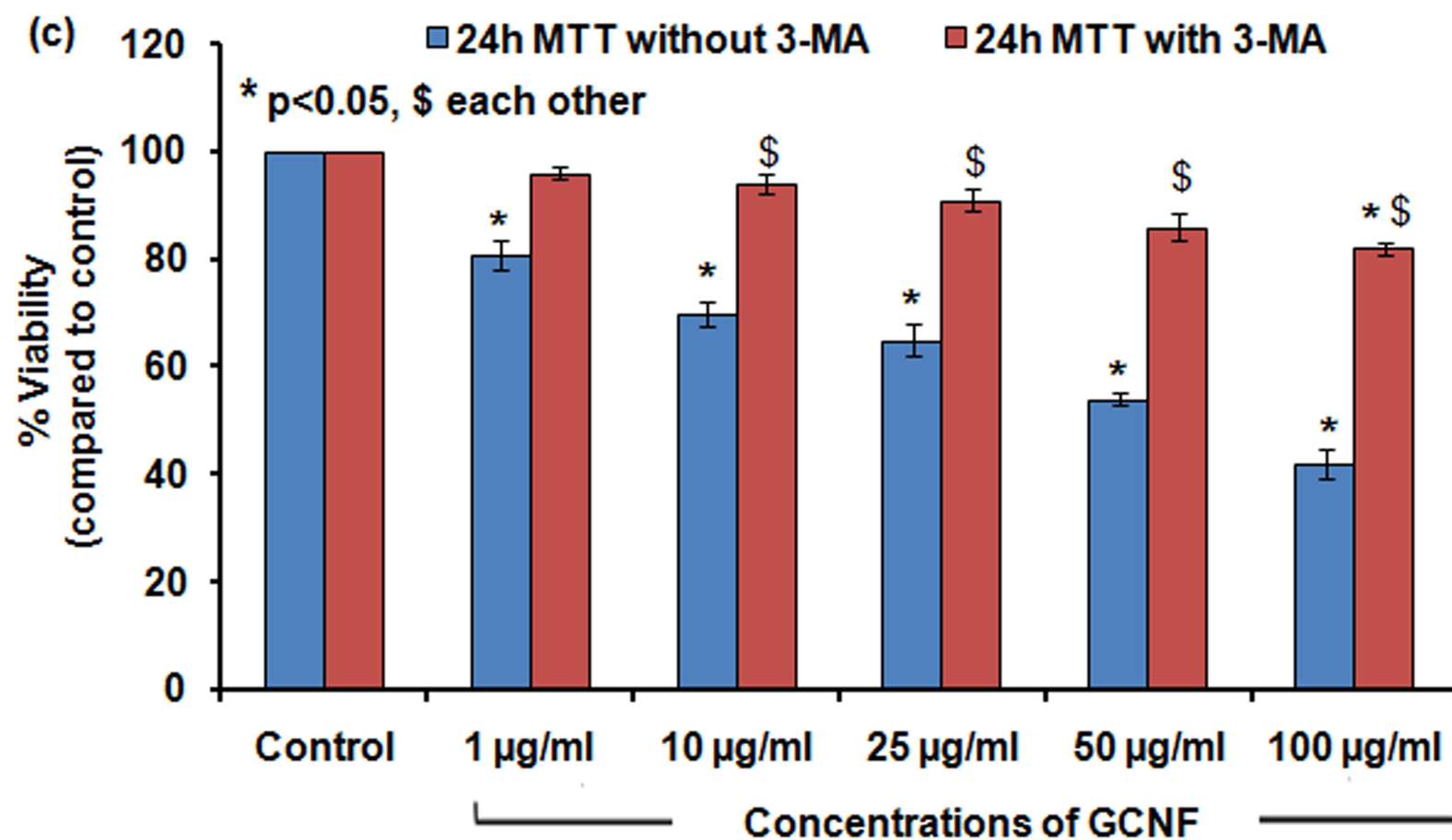

Supplement: Supplementary file 5 — GCNF induced autophagy mediated apoptosis in A549 cells. (a) Immunoblotting and respective densitometry analysis of apoptotic as well as autophagic protein in the presence of 3 – methyladenine (3 - MA) after GCNF (25 μg/ml) exposure in A549 cells. GAPDH was used as loading control. (b) Immunoblotting analysis for specificity of LC siRNA at protein level. (c) Immunoblotting and respective densitometry analysis of apoptotic as well as autophagic protein in the presence of LC3 siRNA after GCNF (25 μg/ml) exposure in A549 cells. GAPDH was used as loading control. Values are expressed as mean ± SE of three independent experiment. *p<0.05 was considered as statistical significant. (d) Viability of GCNF exposed cells was assessed using MTT assay in the presence or absence of 3-MA to check the role of autophagy in cell death. Values are expressed as mean ± SE of three independent experiment. *p<0.05 was considered as statistical significant. (PDF 1330 kb) [file 12989_2017_194_MOESM5_ESM.pdf]

**Figure S6**

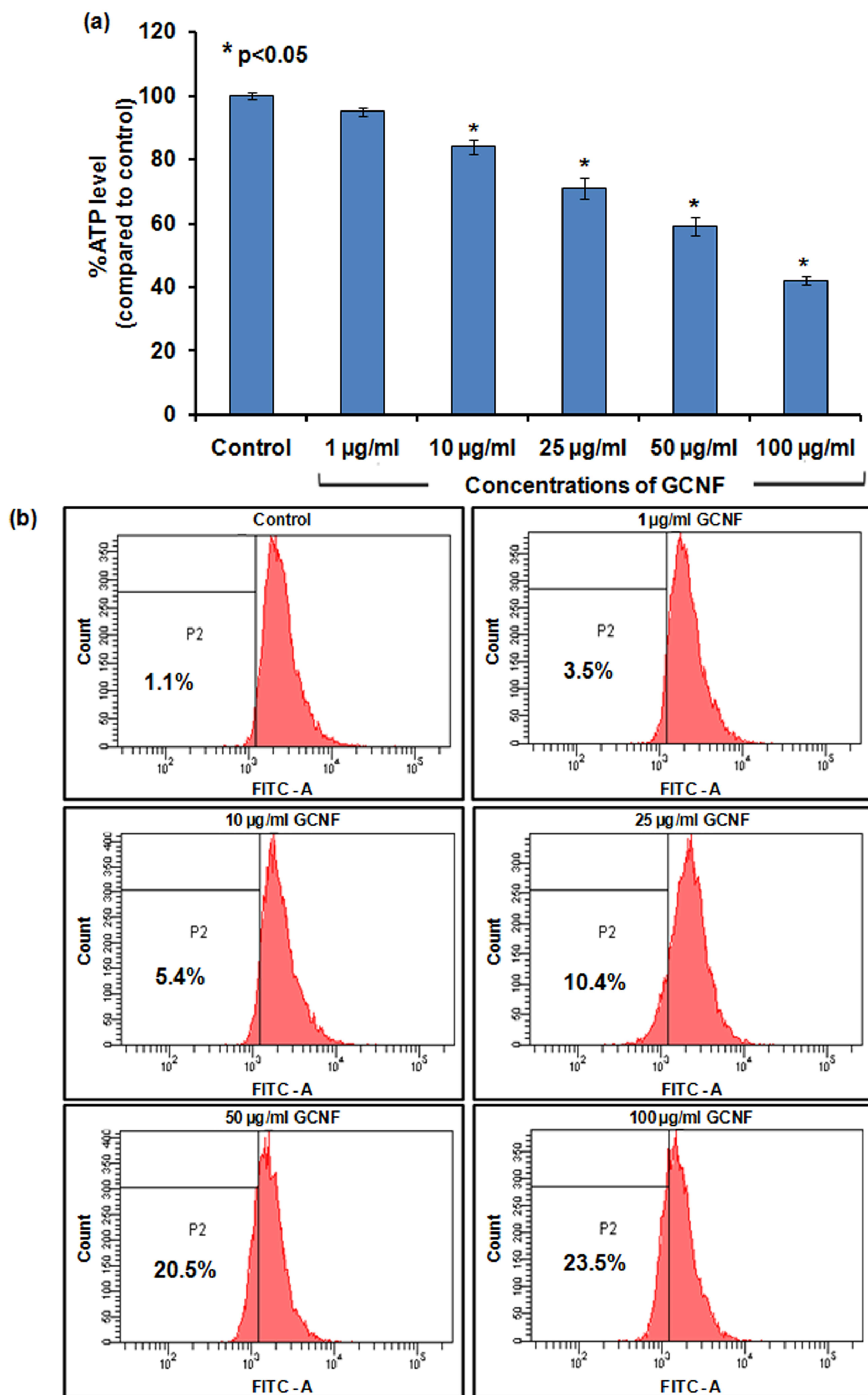

Supplement: Supplementary file 6 — GCNF induced ATP loss and inhibition of glucose uptake in A549 cells. (a) A549 cells exposed to GCNF (1 – 100 μg/ml) for 24 h time period showed a dose dependent decrease in ATP level compared to control cells which was measured using ATP measurement kit by a luminometer. Values are expressed as mean ± SE of three independent experiment. *p<0.05 was considered as statistical significant. (b) A549 cells were treated with GCNF for 24 h time and glucose uptake was determined using 2 - NBDG according to the manufacturer’s instruction. Significant reduction in fluorescence was detected as measured by the flow cytometer. (PDF 913 kb) [file 12989_2017_194_MOESM6_ESM.pdf]

**Figure S7**

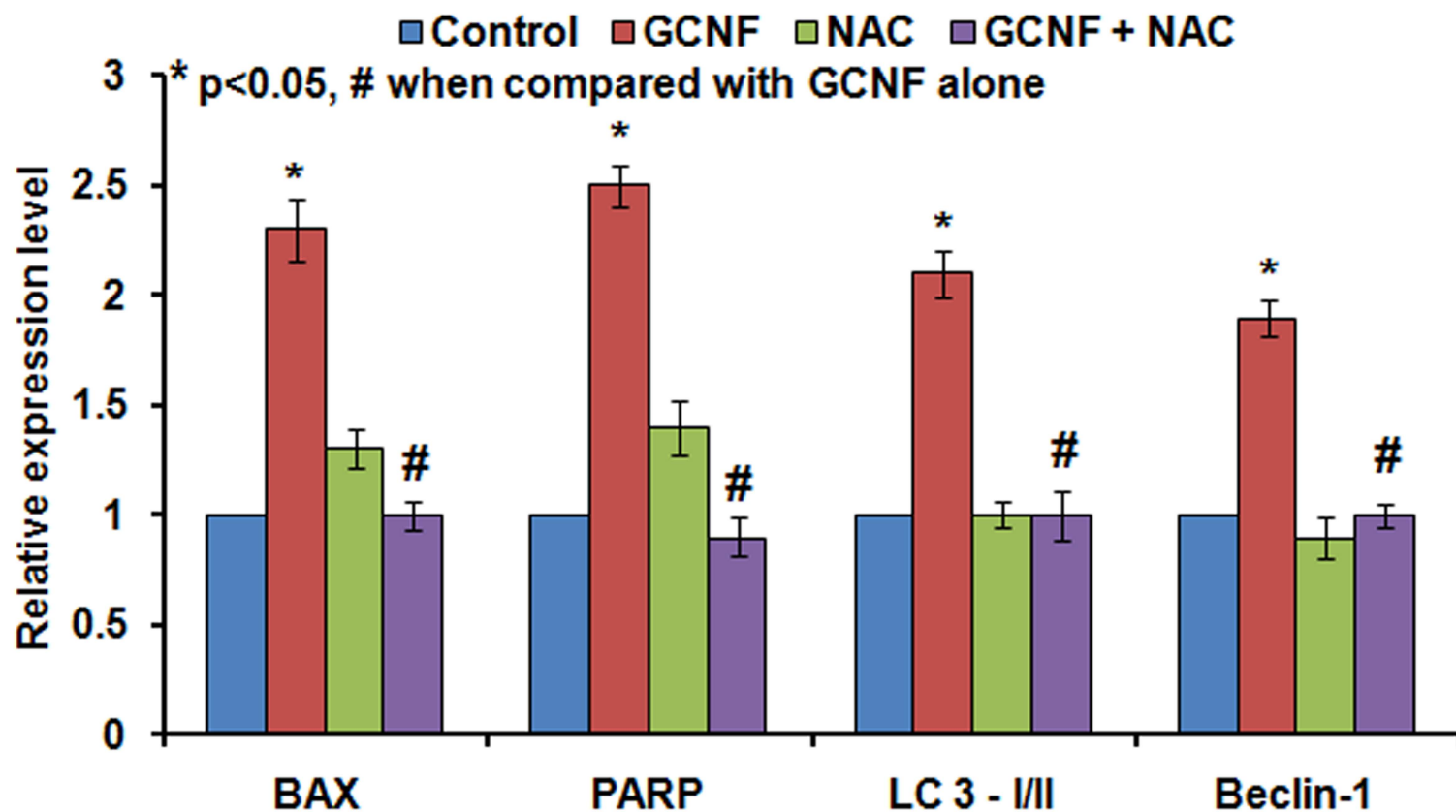

Supplement: Supplementary file 7 — Densitometry analysis of apoptotic as well as autophagic protein in presence and absence of NAC after GCNF (25 μg/ml) exposure in A549 cell. GAPDH was used as an internal control. Values are expressed as mean ± SE of three independent experiment. *p<0.05 was considered as statistical significant. (PDF 290 kb) [file 12989_2017_194_MOESM7_ESM.pdf]

**Figure S8**

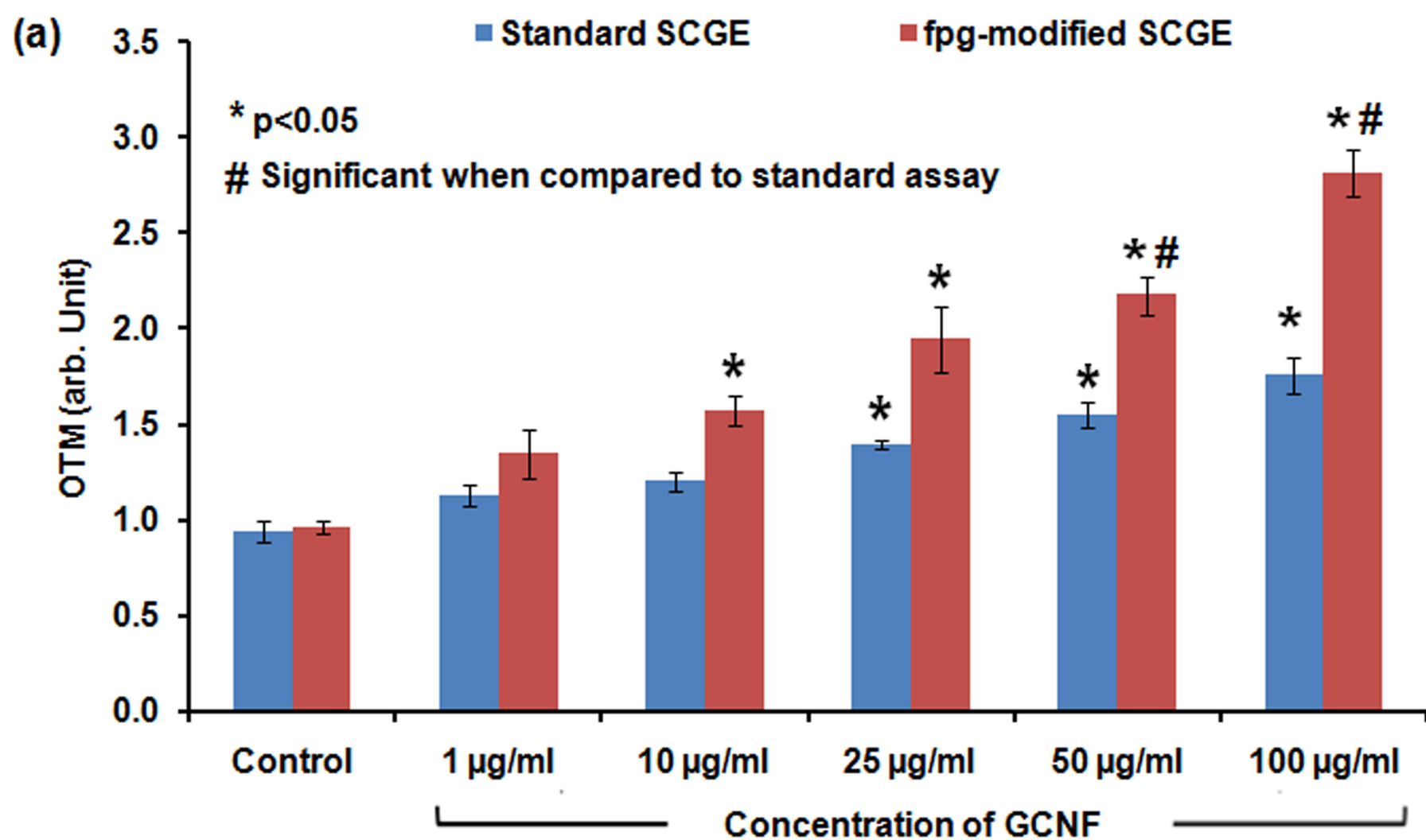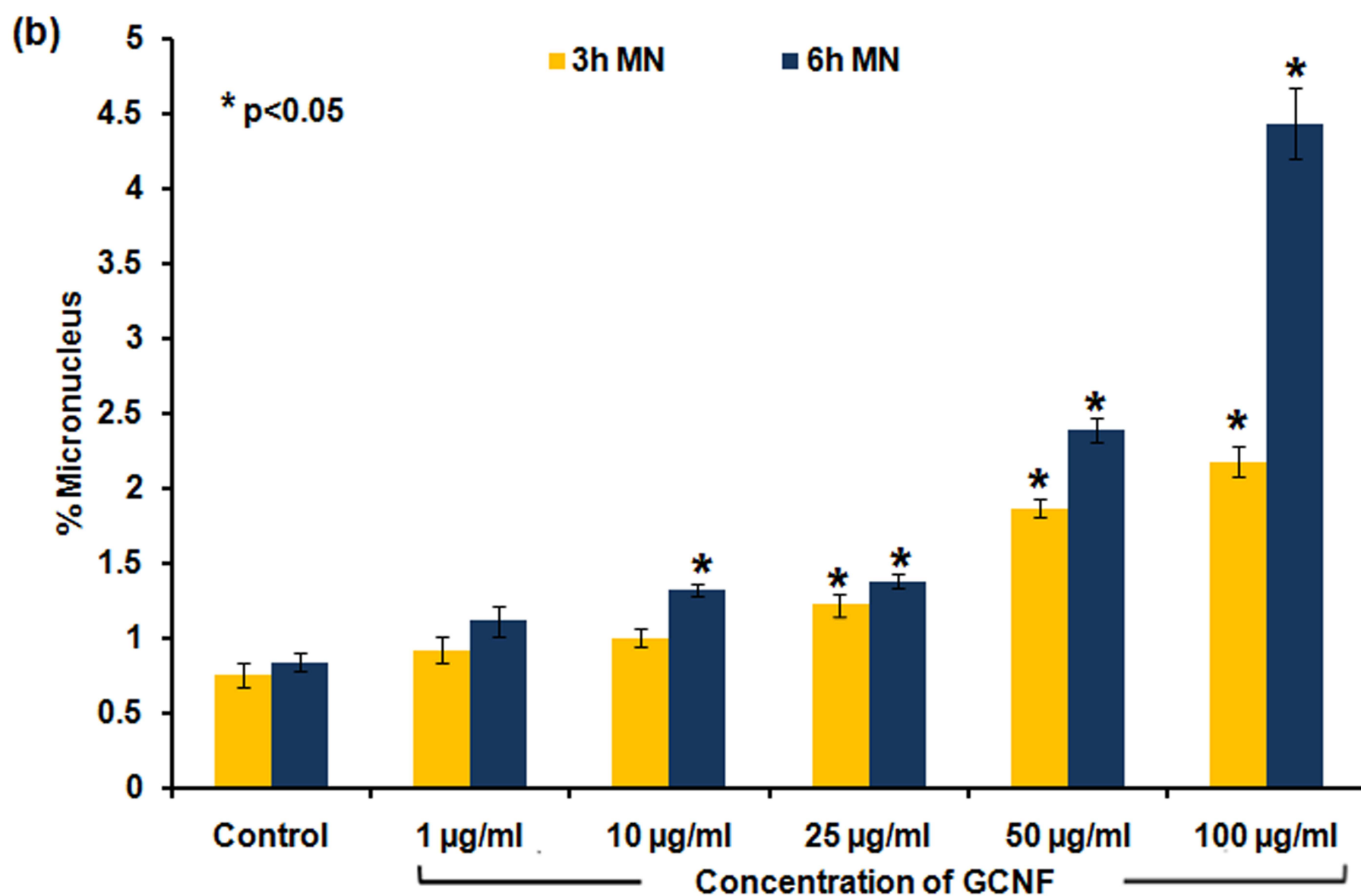

Supplement: Supplementary file 8 — GCNF induced DNA damage and chromosomal breakage in A549 cells. (a) Assessment of DNA damage by GCNF in A549 cells was carried out using Comet assay (Standard as well as Fpg modified) after 6 h of exposure. Results showed a significant increase in OTM value (Comet parameter) of exposed cells compare to control cells. Values are expressed as mean ± SE of three independent experiment. *p<0.05 was considered as statistical significant. # when compared to standard alkaline Comet assay. (b) Flow cytometry based micronucleus (MN) assay showed a significant increase in MN formation with increasing concentration of GCNF after 3 h and 6 h exposure. This depicts the induction of chromosomal breakage in A549 cells after GCNF exposure. Values are expressed as mean ± SE of three independent experiment. *p<0.05 was considered as statistical significant. (PDF 674 kb) [file 12989_2017_194_MOESM8_ESM.pdf]
